# Single-Cell Atlas of the *Drosophila* Leg Disc Identifies a Long Non-Coding RNA in Late Development

**DOI:** 10.3390/ijms23126796

**Published:** 2022-06-18

**Authors:** Joyce Tse, Tsz Ho Li, Jizhou Zhang, Alan Chun Kit Lee, Ivy Lee, Zhe Qu, Xiao Lin, Jerome Hui, Ting-Fung Chan

**Affiliations:** 1 School of Life Sciences, The Chinese University of Hong Kong, Hong Kong; joycekytse@gmail.com (J.T.); andrewli@cuhk.edu.hk (T.H.L.); zjzace@outlook.com (J.Z.); cklee@link.cuhk.edu.hk (A.C.K.L.); ivyleeting@gmail.com (I.L.); quzheouc@gmail.com (Z.Q.); alanlamsiu@gmail.com (X.L.); jeromehui@cuhk.edu.hk (J.H.); 2 State Key Lab of Agrobiotechnology, The Chinese University of Hong Kong, Hong Kong

**Keywords:** *Drosophila*, leg imaginal disc, lncRNA, development, scRNA-seq, scATAC-seq

## Abstract

The *Drosophila* imaginal disc has been an excellent model for the study of developmental gene regulation. In particular, long non-coding RNAs (lncRNAs) have gained widespread attention in recent years due to their important role in gene regulation. Their specific spatiotemporal expressions further support their role in developmental processes and diseases. In this study, we explored the role of a novel lncRNA in *Drosophila* leg development by dissecting and dissociating *w^1118^* third-instar larval third leg (L3) discs into single cells and single nuclei, and performing single-cell RNA-sequencing (scRNA-seq) and single-cell assays for transposase-accessible chromatin (scATAC-seq). Single-cell transcriptomics analysis of the L3 discs across three developmental timepoints revealed different cell types and identified *lncRNA:CR33938* as a distal specific gene with high expression in late development. This was further validated by fluorescence *in-situ* hybridization (FISH). The scATAC-seq results reproduced the single-cell transcriptomics landscape and elucidated the distal cell functions at different timepoints. Furthermore, overexpression of *lncRNA:CR33938* in the S2 cell line increased the expression of leg development genes, further elucidating its potential role in development.

## 1. Introduction

Long non-coding RNAs (lncRNAs) are defined as RNAs longer than 200 nucleotides and not translated into functional proteins. Human GENCODE (v40) identifies 17,748 lncRNA genes, which roughly equates to the number of protein-coding genes (19,988) signifying the importance of lncRNAs. The majority of lncRNAs are transcribed by RNA polymerase II and are often 5′-end 7-methyl guanosine (m7G) capped, 3′-end polyadenylated, and spliced similarly to mRNAs. They are often classified based on their position relative to neighboring genes (divergent, convergent, intergenic, antisense, sense, enhancer, intronic, and miRNA host), transcript length (long intergenic, very long intergenic, and macroRNA), association with annotated protein-coding genes, association with other DNA elements, protein-coding RNA resemblance, association with repeats, association with a biochemical pathway, sequence and structure conservation, biological state, association with subcellular structures, and function [1,2]. As lncRNAs provide supportive roles by fine-tuning gene expression levels at the epigenetic, transcriptional, and post-transcriptional levels, they are implicated in various biological processes and diseases. The contribution of lncRNAs to organ development in several mammalian species has revealed a transition of broadly expressed lncRNAs towards an increasing number of spatiotemporal-specific and condition-specific lncRNAs [3]. The role of lncRNAs in cancer has been studied extensively, but they are also involved in many other human diseases from neurological disorders to cardiovascular issues [4]. Notably, lncRNA expression is generally spatiotemporal specific, indicating the unique functions and probable pharmacological targeting of lncRNA.

*Drosophila melanogaster* (fruit fly) is an ideal model organism to study developmental and cellular processes in higher eukaryotes, including humans, because a wide range of genetic tools can be applied and its genome has been extensively studied [5]. In fact, the *D. melanogaster* genome is 60% homologous to that of humans and nearly 75% of human disease-causing genes are believed to have functional homologs in the fruit fly [6]. Furthermore, its short generation time, high fecundity, and low maintenance as well as the abundance of publicly available fly stocks and databases also make *D. melanogaster* an appealing model organism. 

Despite different taxonomic origins, the *Drosophila* larval leg disc, which develops into the adult leg, is an ideal model for studying the complex vertebrate limb because it is relatively simple and amenable to genetic manipulations. Research on fly imaginal discs has revealed the tissue compartments and organ-specific regulator genes critical to development, and has generated established models for the study of cellular interactions and complex genetic pathways [7]. Moreover, the easy accessibility of imaginal discs further supports their utility.

Advances in the past decade on single-cell RNA sequencing (scRNA-seq) and related computational analysis pipelines have allowed scientists and bioinformaticians to understand the cellular heterogeneity of tissues at an unprecedented level, from manually selecting a single-cell under the microscope to plate-based and droplet-based high throughput methods with multimodal capabilities [8]. Since the publication of the first single-cell transcriptome study based on a next-generation sequencing platform, the number of publications on scRNA-seq addressing development, disease, and bioinformatics tool improvement has exponentially grown [9,10,11,12]. Many of these publications have focused on developmental biology, often involving single-cell studies, as it represents a crucial period during which cells first begin to differentiate [13]. Single-cell transcriptomics studies on *Drosophila* larval imaginal wing and eye-antennae discs have emerged since 2018 [14,15,16,17,18,19] and shown that single cells could be mapped to the distinct subregions of their respective imaginal discs, thus confirming the spatial expression of genes determined by previous immunostaining methods. 

While the Fly Cell Atlas recently performed single-nucleus RNA-sequencing (snRNA-seq) on adult *Drosophila* legs [20], single-cell transcriptomics and epigenomics studies on the developing leg imaginal disc remain lacking due to the challenges of its dissection compared to the larger wing and eye-antennae discs. We thus report the single-cell transcriptomic and epigenomic landscapes of *w^1118^* third leg discs (L3) across three time points of development of third-instar larvae. We identified and validated a novel, highly expressed lncRNA in the distal epithelial cells that changes its spatial expression at various stages of development and confirms its importance in leg development.

## 2. Results

### 2.1. Generation of a Transcriptomic Cell Atlas of the Developing Leg Imaginal Disc

#### 2.1.1. Single-Cell RNA-Sequencing Identifies Four Main Cell Types in L3 Discs

**Figure 1:**
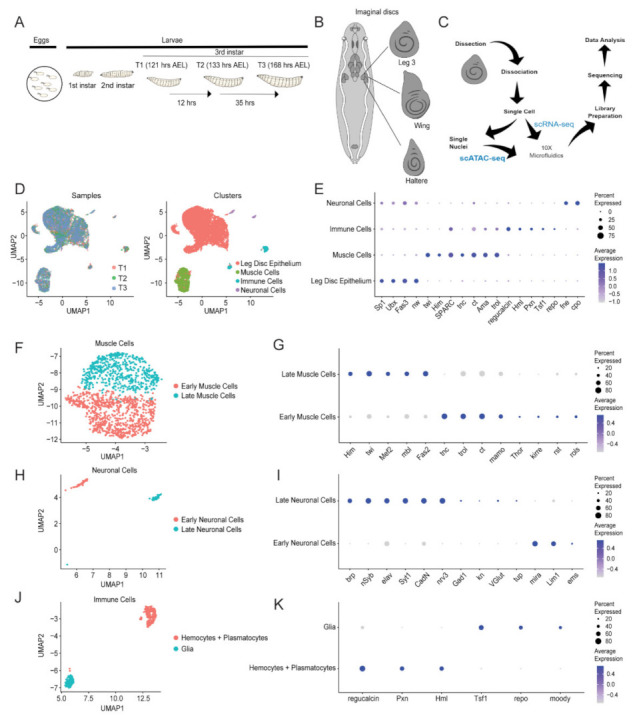
**scRNA-seq revealed the major cell types of *D.melanogaster* third-instar third leg discs.** (**A**) The time course of leg disc dissection. Embryos grew until dissection at T1 (121 h AEL), T2 (133 h AEL) or T3 (168 h AEL). (**B**) The third leg disc was differentiated from other leg discs as it occurred as a mid-size disc with a concentric ring-like pattern at its center within a trio of discs on bilateral sides of the larvae which also included the wing and haltere discs. (**C**) Flowchart of scRNA-seq and scATAC-seq experiments. Dissected leg discs were dissociated into single cells, a portion of which were used for scRNA-seq with the remaining cells having their nuclei isolated for scATAC-seq. Both scRNA-seq and scATAC-seq used the 10× Genomics Chromium Controller and proceeded with their respective library preparation protocols, sequencing, and data analysis. (**D**) UMAP visualizations of the scRNA-seq data show that T1, T2, and T3 overlay each other, although the four identified cell types were quite segregated. (**E**) Dot plot showing the known marker genes of the respective cell types identified in the UMAP visualization. (**F**) Muscle cell subset of the scRNA-seq data showing differentiation between early and late muscle cells. (**G**) Dot plot showing the known marker genes of the early and late muscle cells identified in the UMAP visualization. (**H**) Neuronal cell subset of the scRNA-seq data showing differentiation between early and late neuronal cells. (**I**) Dot plot showing the known marker genes of the early and late neuronal cells identified in the UMAP visualization. (**J**) Immune cell subset of the scRNA-seq data showing differentiation between hemocytes (and plasmatocytes) and glia. (**K**) Dot plot showing the marker genes of hemocytes (and plasmatocytes) and glia identified in the UMAP visualization.

To study the cellular heterogeneity of developing L3 discs, collected embryos were dissected for L3 discs at 121 h (T1), 133 h (T2), and 168 h (T3) after egg laying (AEL) (Figure 1A) for scRNA-seq. Sequencing statistics showed similar data quality amongst the three samples, including the percentage of mapped reads, percentage of mapped reads aligned to genes, number of cells, and mean reads per cell (Table S1). The L3 disc was identified as a trio of discs on either side of the larval body that differed from the wing and haltere discs in morphology and patterning (Figure 1B). Cell preparation workflow involved dissection and dissociation of L3 discs into single cells, after which a portion of the cells was used for scRNA-seq and the remaining cells having their nuclei isolated for single-cell assay for transposase-accessible chromatin (scATAC-seq) (Figure 1C). Both assays used the 10× Genomics platform and the prepared libraries were subjected to sequencing and subsequent data analysis. The integrated dataset overlayed T1, T2, and T3 individual samples and identified four distinct clusters (Figure 1D). The largest cluster represented the leg disc epithelium, which expressed epithelial markers *Fasciclin 3* (*Fas3*) and *narrow* (*nw*) (Figure 1E) [21]. Expression of *Sp1* and *Ultrabithorax* (*Ubx*) confirmed that the cells originated from L3 discs [22,23]. The second-largest cluster represented muscle cells, which expressed the muscle markers *twist* (*twi*), *Holes in muscle* (*Him*), *Secreted protein*, *acidic*, *cysteine-rich* (*SPARC*), *tenectin* (*tnc*), *cut* (*ct*), *Amalgam* (*Ama*), and *terribly reduced optic lobes* (*trol*) [17,24,25]. The identity of the immune cell cluster was determined by the expression of *regucalcin*, *Hemolectin* (*Hml*), *Peroxidasin* (*Pxn*), *Transferrin 1* (*Tsf1*), and *reversed polarity* (*repo*) [19,26,27]. The smallest cluster represented the neuronal cells, which expressed *found in neurons* (*fne*) and *couch potato* (*cpo*) [15,28]. The relative expression levels of the marker genes were tabulated (Table A1).

#### 2.1.2. Subclustering of the Main Cell Types Reveals Cell Subtypes

The muscle cell cluster was composed of early and late muscle cell subclusters (Figure 1F). The early cells expressed *tenectin* (*tnc*), *terribly reduced optic lobes* (*trol*), *cut* (*ct*), *maternal gene required for meiosis* (*mamo*), *Thor*, *kin of irre* (*kirre*), *roughest* (*rst*), and *rolling pebbles* (*rols*) (Figure 1G) [17,19,24,25,29]. The late cells expressed *Holes in muscle* (*Him*), *twist* (*twi*), *Myocyte enhancer factor 2* (*Mef2*), *muscleblind* (*mbl*), and *Fasciclin 2* (*Fas2*) [19,30]. Early muscle cells increased expression of late muscle cell marker *Fas2* over time in terms of both expression level and the number of cells that expressed this gene (Figure S1). Late muscle cell marker *Mef2*, a skeletal muscle differentiation transcription factor, similarly increased expression in the late muscle cell subcluster over time in terms of both expression level and the number of cells that expressed the gene. The heatmap of the most upregulated genes in the early and late muscle cells showed a distinction in upregulated genes between the two subclusters (Figure S2).

The neuronal cell cluster was also composed of early and late neuronal cell subclusters (Figure 1H). The early cells expressed *miranda* (*mira*), *LIM homeobox 1* (*Lim1*), and *empty spiracles* (*ems*) (Figure 1I) [31,32,33]. The late cells expressed *bruchpilot* (*brp*), *neuronal Synaptobrevin* (*nSyb*), *embryonic lethal abnormal vision* (*elav*), *Synaptotagmin 1* (*Syt1*), *Cadherin-N* (*CadN*), *nervana 3* (*nrv3*), *Glutamic acid decarboxylase 1* (*Gad1*), *knot* (*kn*), *vesicular glutamate transporter* (*VGlut*), and *tailup* (*tup*) [34,35,36,37]. The heatmap of the most upregulated genes in the early and late neuronal cells showed a clar distinction between the two subclusters (Figure S3).

The immune cell cluster was composed of glia and hemocytes (including plasmatocytes), which are the phagocytes found in invertebrates **(**Figure 1J). Glial cells expressed *Transferrin 1* (*Tsf1*), *reversed polarity* (*repo*), and *moody* [27,38], while the hemocytes and plasmatocytes expressed *regucalcin*, *Peroxidasin* (*Pxn*), and *Hemolectin* (*Hml*) (Figure 1K) [19,26]. These markers were highly specific to their respective cell subtypes and the heatmap of the most upregulated genes in the glia and hemocytes (including plasmatocytes) showed a clear distinction between the two subclusters (Figure S4).

**Figure 2:**
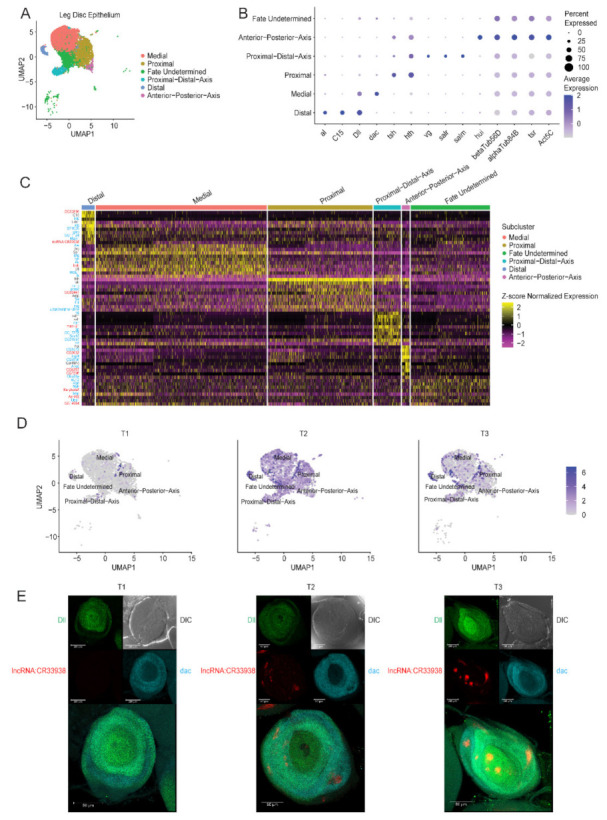
**Subclustering of the epithelial cell cluster with identified cell subtypes along the PD axis and a distal-specific *lncRNA:CR33938*.** (**A**) Leg disc epithelium subset of the scRNA-seq data showing differentiation of cells along the PD axis of the fly leg. (**B**) Dot plot showing the known marker genes of the proximal, medial, and distal cells as well as the earlier stem-cell like cells of the PD axis. (**C**) Heatmap of the top ten most upregulated genes for each cell subtype (subcluster) of the epithelial cell cluster, where black represents known marker genes, blue represents genes of known function as potential markers, and red represents genes of unknown functions. *LncRNA:CR33938* was identified as one of the most upregulated genes in the distal cells. (**D**) Feature plots showing the expression levels of *lncRNA:CR33938* in different epithelial subclusters across T1, T2, and T3. (**E**) Validation of the scRNA-seq *lncRNA:CR33938* identified using FISH showing negligible expression during T1, epithelium wide expression in T2, and mainly distal-specific expression in T3. Scale bar represents 50 µm.

The leg disc epithelium cluster was subclustered into six cell subtypes, including the distal, medial, and proximal cells as well as stem cell-like cells, such as those of the proximal-distal-axis (PD axis) and anterior-posterior-axis (AP axis), and cells of undetermined fate (Figure 2A). Identification of fate undetermined cells was based on their top upregulated DEGs and gene ontology analysis of the DEGs (Table A2 and Table A3). The distal cells expressed the markers *aristaless* (*al*), *C15*, and *Distal-less* (*Dll*) (Figure 2B) [22]. The medial cells expressed *dachshund* (*dac*) and *Dll*, while the proximal cells expressed *teashirt* (*tsh*) and *homothorax* (*hth*) [22]. The PD axis cells expressed *vestigial* (*vg*), *spalt-related* (*salr*), and *spalt major* (*salm*) [39], and the AP axis cells expressed *hase und igel* (*hui*) (FlyBase ID FBgn0033968).

### 2.2. Identification and Characterization of a Novel Long Non-Coding RNA

#### 2.2.1. Identification of a Long Non-Coding RNA of Unknown Function in Distal Cells

The most upregulated genes in each leg disc epithelium subcluster are shown in a heatmap (Figure 2C). The genes colored black represent known markers for their respective subclusters, those colored blue represent genes with known functions as potential markers for their respective subclusters, and the genes colored red represent genes with unknown functions as potential markers for their respective subclusters. lncRNA (*lncRNA:CR33938*) is unique because the 10× 3′ gene expression kit uses oligo(dT) primers to detect polyA-tailed transcripts, which mostly include mRNAs. However, *lncRNA:CR33938* expression was observed in this study (Figure 2D). Indeed, *lncRNA:CR33938* was identified in other studies by using polyA^+^ bulk RNA-sequencing [40,41]. Upon splitting the integrated data into its respective samples (T1, T2, and T3), *lncRNA:CR33938* expression was negligible in T1, appeared more widespread in T2 and became specific to the distal cells in T3. Note that while most distal cells expressed *lncRNA:CR33938*, a small subset of medial and proximal cells also expressed the lncRNA.

#### 2.2.2. Experimental Validation of lncRNA:CR33938 Expression in L3 Discs

Fluorescence *in-situ* hybridization (FISH) of *lncRNA:CR33938* in T1, T2 and T3 L3 discs was performed alongside region-delineating controls *Dll* and *dac* (Figure 2E). Expression of only *Dll* represented the distal cells, while co-expression of *Dll* and *dac* or only *dac* represented the medial cells. *LncRNA:CR33938* expression in T1 L3 discs did not occur. *LncRNA:CR33938* expression in T2 L3 discs was present in the proximal, medial, and distal cells, while *lncRNA:CR33938* expression in T3 L3 discs was most prominent in distal cells, although medial cells also showed more limited expression. More specifically, six regions of large punctated *lncRNA:CR33938* expression were observed in T3, including four regions of high expression and two regions of lower expression. Three of the six regions were within the distal cells, while the other three regions were outside of the distal cells, suggesting expression of *lncRNA:CR33938* in cells other than the distal cells, namely the medial cells. The proximal cells also had a low level of *lncRNA:CR33938* expression, as displayed by a tint of red fluorescence peripheral to the medial cells delineated by the *dac* marker. In fact, Figure 2D did show other cells expressed the lncRNA, but these cells represented only small subsets of the subclusters. Thus, these FISH results corroborated the scRNA-seq data. Note that the punctated regional expressions may suggest localized *lncRNA:CR33938* function in aggregates.

#### 2.2.3. Conservation of lncRNA:CR33938 in Insect Species

**Figure 3:**
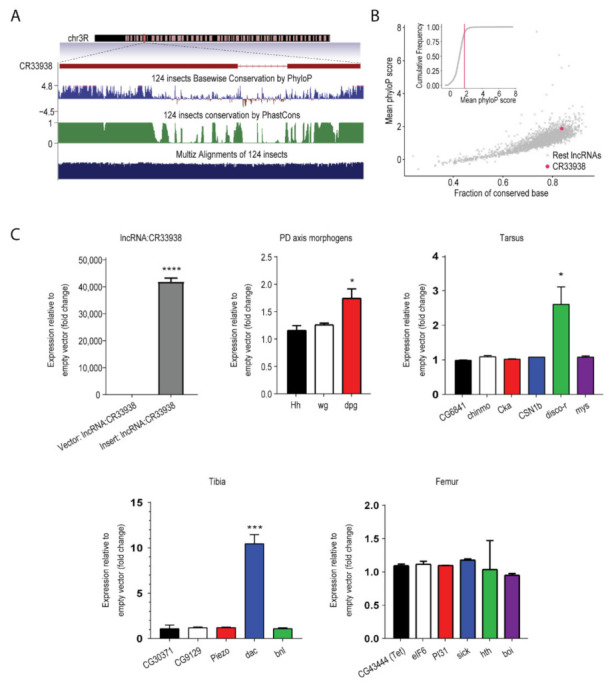
***LncRNA:CR33938* is conserved among insects and overexpression in *Drosophila* S2 cells increased expression levels of genes involved in leg development.** (**A**) *lncRNA:CR33938*, identified on chromosome 3, is conserved within insects. (**B**) The fraction of conserved bases of *lncRNA:CR33938* across insects is greater than 0.8. (**C**) Overexpression of *lncRNA:CR33938* in S2 cells produced an increase in the expression of leg development genes, including PD axis genes, distal leg tarsal *disco-r*, and medial leg tibial *dac* according to qPCR. There was no effect on proximal leg femur genes. *, ***, **** equate to *p*-values of less than 0.05, 0.001 and 0.0001, respectively.

The conservation state of *lncRNA:CR33938* across 124 insect species revealed that the lncRNA had a high conservation level in exon regions (Figure 3A). Moreover, a comparison with the conservation state of all 2258 lncRNAs annotated in the reference annotation suggested that *lncRNA:CR33938* was more conserved than 90% of the other lncRNAs (Figure 3B). These concordances reflected a critical regulatory role of *lncRNA:CR33938* in insect development.

#### 2.2.4. Overexpression of lncRNA:CR33938 in S2 Cells

Transient overexpression of full-length *lncRNA:CR33938* in S2 cells produced an approximately 40,000-fold increase in expression level compared to the empty vector control (Figure 3C) according to qRT-PCR. Correspondingly, expression of the PD axis genes (*Hh*, *wg*, and *dpp*) showed an increasing trend upon *lncRNA:CR33938* overexpression. While there was no effect on the expression of genes controlling proximal leg femur growth, expression of distal leg tarsal *disco-r* and medial leg tibial *dac* significantly increased with *lncRNA:CR33938* overexpression. This corroborated the scRNA-seq and FISH data that *lncRNA:CR33938* more greatly affected (and was normally expressed in) the distal end of the leg.

### 2.3. Generation of an Epigenomic Cell Atlas of the Developing Leg Imaginal Disc

#### 2.3.1. scATAC-seq Identified Similar Cell Types as scRNA-seq

**Figure 4:**
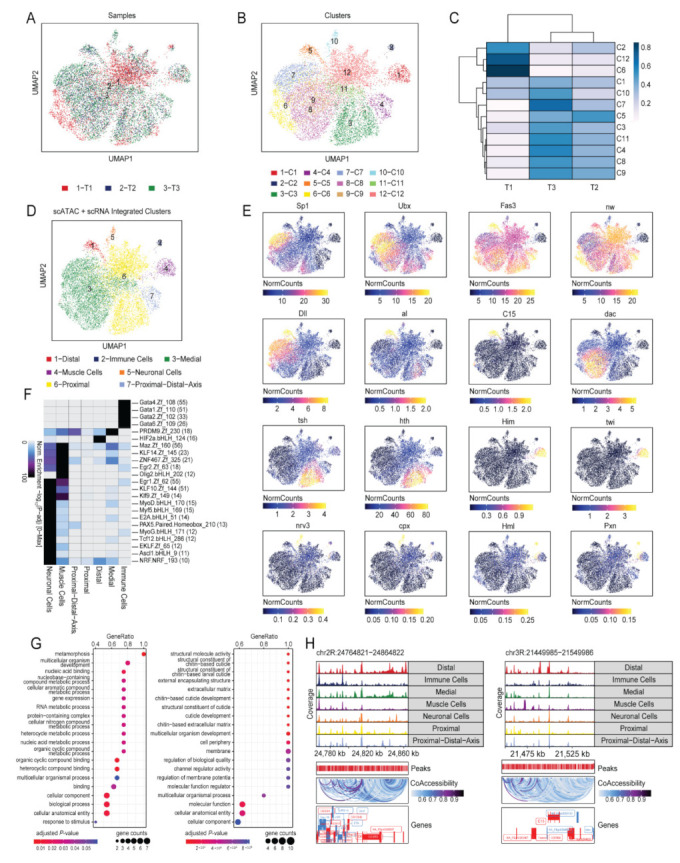
**scATAC-seq revealed the same major cell types of *Drosophila* L3 disc as in scRNA-seq.** (**A**) UMAP visualization of the scATAC-seq data, showing that T1, T2, and T3 overlay one another. (**B**) UMAP visualization revealing the different clusters identified prior to integration with scRNA-seq. (**C**) Heatmap showing the proportion of cells of each cluster within each sample (T1, T2, and T3). The color scale represents the cell proportion within each cluster. (**D**) UMAP visualization of clusters after integration of scATAC-seq data with scRNA-seq data. Most cell types and cell subtypes were remapped, including the proximal, medial, and distal cells as well as the muscle, neuronal, immune, and stem-cell like cells of the PD axis. (**E**) Feature plots showing the known marker genes, and the respective cell types and cell subtypes identified in the UMAP visualization after scRNA-seq data integration. (**F**) Heatmap of important motifs in each cluster. (**G**) Gene Ontology analysis of the chromatin-accessible distal genes of T2 and T3 relative to those of T1 showed many metabolic processes occurring in early T2 (left), while many chitin-based cuticle development processes occurred in late T3 (right). (**H**) Genome tracks of distal marker genes (*Dll* and *C15*) revealed high co-accessibility in neighboring genes.

The sample-wide integrated scATAC-seq dataset showed an overlaying of the T1, T2, and T3 individual samples (Figure 4A) and identified twelve distinct clusters based on differences in chromatin accessibility (Figure 4B). A heatmap distinguished the proportion of cells in each cluster at each timepoint and showed differences in chromatin accessibility and cell composition across three timepoints (Figure 4C). For example, cluster 6 (C6) showed greater than 80% of the cells in T1, a small proportion of the cells in T2, and nearly no cells in T3. Similarly, T1 had many cells from cluster 12 (C12) and cluster 2 (C2).

Upon integration of the chromatin accessibility data with the gene expression data, seven cell types identified in scRNA-seq were transferred to the scATAC-seq clusters (Figure 4D). These cell types corresponded to the cell subtypes of the PD axis of the leg disc epithelium (proximal, medial, and distal cells) as well as those of the muscle, neuronal, and immune cells.

The cell type identities were confirmed by an inferred gene score of chromatin accessibility for a list of known marker genes specific to the cell types (Figure 4E). Similar to the gene expression data in scRNA-seq, high gene scores of *Sp1* and *Ubx* confirmed that the cells originated from L3 discs. All cells that composed the leg disc epithelium (proximal, medial, and distal cells) showed markers *Fas3* and *nw*. The presence of *Dll* only (without *dac*), *al*, and *C15* confirmed the identity of the distal cells. *Dll* (with *dac*) and *dac* only confirmed the identity of the medial cells. Similarly, *tsh* and *hth* were markers for the proximal cells, while *Him* and *twi* represented the muscle cells. The neuronal cells showed high gene scores for *nervana 3* (*nrv3*) and *complexin* (*cpx*), and the immune cells produced high scores for *Hml* (for hemocytes) and *Pxn* (for plasmatocytes) markers.

The most enriched motifs for each cell type are shown as a heatmap (Figure 4F). The GATA motifs were evident in the immune cells, with several GATA family members observed. The PRDM9 and HIF2a.bHLH motifs were highly enriched in the medial and distal cells, respectively. While the NRF motif was enriched in the proximal cells, its enrichment was more evident in the neuronal cells. The muscle cells were enriched in many motifs, including Maz, KLF14, ZNF, Egr2, Olig2, Egr1, KLF10, and Klf9. The neuronal cells were also enriched for many motifs, including MyoD, Myf5, E2A, PAX5, MyoG, Tcf12, EKLF, Ascl1, and NRF.

#### 2.3.2. Chromatin Accessibility Differentiated the T1, T2, and T3 Distal Cell Functions

Gene set enrichment of T2 genes relative to T1 and T3 genes relative to T1 showed differences in cellular processes (Figure 4G). The T2 distal cells were more involved in metabolic processes, while the T3 distal cells had a larger role in chitin-based larval cuticle development.

Fragment coverage within the 40,000 base pairs on either side of the distal cell marker gene *Dll* showed increased coverage in the distal cells with marked co-accessibility in neighboring genes (Figure 4H). Distal cell marker gene *C15* similarly displayed increased coverage in the distal cells with marked co-accessibility in neighboring genes.

## 3. Discussion

We used scRNA-seq and scATAC-seq to explore the *Drosophila* L3 disc transcriptomic and epigenomic landscapes, respectively, at three timepoints of development. The multi-omics datasets corroborated each other and showed similar cell types that delineated the various regions of the leg disc, namely, those along the PD axis. Moreover, scRNA-seq identified an experimentally validated late-stage distal-specific and conserved lncRNA (*lncRNA:CR33938*) that, upon further characterization by overexpression studies, promoted distal leg growth gene expression. In addition, differences in chromatin accessibility determined by scATAC-seq indicated the disparate functions of early- and late-stage distal cells.

Given that the three legs of *Drosophila* differ in their developmental programs, their underlying differences cannot be ignored when studying leg disc development [23]. Subsequently, we specifically isolated the third leg disc to provide a more coherent single-cell atlas.

Simultaneous multi-omics library preparation methods, where the same cell or nuclei are used for different single-cell assays, were not available at the time these experiments were completed. As a result, the same cell suspension was used for both scRNA-seq and scATAC-seq to minimize biological variation. Furthermore, the limited number of cells extracted per leg disc prevented the execution of multiple experiments of biological replicates, in which one experiment consisted of one replicate. Rather, a single assay comprised of many biological replicates was conducted for each time point.

The computationally determined assignment of cell types to clusters depended upon the most upregulated genes in each cluster and prior information about cell type-specific marker genes. In addition to the prominent distal, medial, and proximal cell types, cells that did not express explicit marker genes denoting specific cell types represented early developing cells with undetermined fate, which we referred to as “stem-cell like cells”.

We found a large cluster of epithelial cells and a smaller cluster of muscle cells in the L3 discs, which corroborated previous studies that have shown the presence of many epithelial cells and accompanying muscle cells in the wing discs of third-instar larva [15,16,17]. Previous studies have suggested that the epithelial cells of the wing disc can be mapped to distinct subregions, including the pouch, hinge, notum, and peripodial membrane [16]. Similarly, the epithelial cells of the leg disc could be mapped to distinct proximal, medial, and distal subregions. Regarding muscle cells, research has shown that they can be subcategorized into direct and indirect flight muscles [17]. Given this finding, we also subcategorized the L3 disc muscle cells based on early versus late muscle development genes.

Our results also demonstrated the presence of neuronal and immune cells in L3 discs, which corroborates the recent single-nucleus transcriptomics study on the adult fruit fly leg by the Fly Cell Atlas showing the presence of various differentiated neurons as well as hemocytes and glial cells [20]. This illustrated that the neuronal cells in the developing leg disc have not yet differentiated, though they can subsequently differentiate.

Despite the publication of several works on single-cell transcriptomic landscapes of the *Drosophila* wing and eye-antennae imaginal discs [14,15,16,17,18,19], this study is the first to describe the transcriptomic and epigenomic landscape of the leg disc, specifically the third leg disc, at single-cell resolution. The Fly Cell Atlas study determined the single-nucleus transcriptomic atlas of the adult fruit fly leg, but it was based on fully differentiated tissues [20]. Conversely, our work was based on developing tissue and characterized the importance of an identified lncRNA.

lncRNAs tend to have lower expression levels than protein-coding genes [42]. The detection of *lncRNA:CR33938* by our polyA-tailed single-cell transcriptomics assay indicated that it had a robust level of expression and suggested that it had an important physiological function in leg development given that lncRNA expression is environment-specific [42].

Our work highlighted the spatiotemporal expression of *lncRNA:CR33938.* It was largely distal-specific, as suggested by the scRNA-seq and FISH results, despite some cells other than the distal cells also showing expression. We also provided evidence that *lncRNA:CR33938* may have an important role in leg development. We used a previously published list of larval stage genes that establish the PD axis of the *Drosophila* leg [43]. *LncRNA:CR33938* promoted tarsal leg growth gene expression upregulation, namely *disco-r*. The expression of its paralog, *disco*, is maintained by *Dll*, and *disco* gene function is also required for the maintenance of *Dll* expression [44]. Given the important function of *disco* in maintaining a key gene in PD axis development, this suggests that *lncRNA:CR33938* has an important potential role in leg development. Note that *dac* was also upregulated by *lncRNA:CR33938* overexpression. This was not surprising, as the lncRNA was also expressed in the medial cells in T2. This suggests that *lncRNA:CR33938* exhibits a spatiotemporal role, in which it first modulates medial leg cell fate early in development and then distal leg cell fate modulation later in development. This hypothesis could be tested by using the GAL80 temperature-sensitive and GAL4 with UAS system of flies to spatiotemporally overexpress *lncRNA:CR33938* and to assess leg phenotype, such as leg length. Prior to this study, *lncRNA:CR33938* did not have an annotated function, but our study indicated that it may influence late-stage distal, and perhaps mid-stage medial, leg growth.

## 4. Materials and Methods

### 4.1. Fly Maintenance and Stocks

The *w^1118^ Drosophila melanogaster* fly line was obtained as a gift from Prof. Edwin Chan’s lab at The Chinese University of Hong Kong. All flies were maintained at room temperature in regular light-dark cycles in vials containing standard cornmeal agar medium (Nutri-fly, #66-112).

### 4.2. Fly Breeding Schedule for T1, T2, and T3

Male and female *w^1118^* flies were allowed to mate for 2 h at room temperature in a clear plastic cup with an attached petri dish containing apple juice agar. After this time had elapsed, embryos were transferred from the apple juice agar plate to a vial containing standard cornmeal agar medium. They were then allowed to grow for 121, 133 or 168 h, corresponding to T1, T2, and T3, after which the third leg discs of these third-instar larvae (L3) were dissected.

### 4.3. Third Leg Disc Dissection and Single Cell Dissociation

At least 70 L3 discs were dissected for T1, and at least 50 L3 discs each were dissected for T2 and T3. These discs were collected in an Eppendorf tube containing phosphate-buffered saline (PBS) with 0.04% bovine serum albumin (BSA) on ice. After pipetting out the PBS from briefly centrifuged samples, we added TrypLE Select Enzyme (10×) (ThermoFisher, Waltham, MA, USA, #A1217702). The discs were then incubated in a thermomixer shaken at 500 rpm for 25 min at 37 °C (with the tube being flicked every five minutes). S2 medium (Gibco, Waltham, MA, USA, #21720) supplemented with 10% fetal bovine serum and 2% penicillin/streptomycin were then added to stop the dissociation reaction. Finally, the isolated single cells were washed and resuspended in PBS + 0.04% BSA.

### 4.4. DNA Library Preparation and Sequencing

The complementary DNA (cDNA) libraries for T1, T2, and T3 were prepared according to the 3′ scRNA-seq library preparation protocol (v3.1) of 10× Genomics. In summary, a microfluidics chip was used to produce GEMs (Gel Bead-in-Emulsions), which are droplets that each contain a single microbead with attached oligonucleotides that include a unique cell barcode, a single cell, and reverse transcription reagents. When the single cell lyses within the intact GEM, the cellular polyA-tailed transcript sequences become exposed, reverse transcription occurs, and each cDNA transcript within the same cell receives the same cell barcode with a different UMI (unique molecular identifier). Subsequently, the droplets lyse and the cell-barcoded cDNA from all cells are pooled and amplified. cDNA library construction involved fragmentation, end-repair, A-tailing, double-sided size selection, and sample index incorporation. Quality control and qualitative analysis of the final library were performed on an Agilent Bioanalyzer DNA High Sensitivity chip (Beijing, China). Sequencing of the libraries was completed on the Illumina NovaSeq6000 platform by Novogene (Beijing, China).

### 4.5. scRNA-seq Raw Data Processing, Quality Assessment, and Filtering

The raw paired-end sequencing data files (Fastq) were processed using the Cell Ranger pipeline v4.0.0 with default settings. Read alignment and UMI counts were based on a BDGP6 genome reference fasta file and annotated by a BDGP6.28 gtf file developed by Ensembl. Cell-UMI count tables were loaded into Seurat v4.0 [45]. Cells with 1000–250,000 UMI counts and less than 5% mitochondrial genes were used as filtering gates to select cells for downstream analysis. We only kept genes with at least 20 UMI counts in all cells. Qualimap (v2.2.1) was further used to assess the percentage of mapped reads and percentage of mapped reads aligned to genes for comparison between T1, T2, and T3.

### 4.6. scRNA-seq Data Integration, Clustering, and Cell Type Identification

The T1, T2 and T3 filtered single-cell datasets were merged and integrated using Seurat (v4.0.) Batch effects between samples were corrected using Harmony (v1.0) prior to clustering analysis. PCA was used to determine the optimal dimension for dimensionality reduction, and clustering was performed based on K-nearest neighbor (KNN) graphs with a resolution of 0.02 before UMAP visualization of the single-cell data in two dimensions. The major cell types of the clusters were identified based on known marker genes, and these marker genes were listed among the most upregulated differentially expressed genes compared to other clusters. All clusters were further subclustered into constituent cells based on known marker genes. Dotplots, featureplots, heatmaps, and UMAPs were then generated. Other than the known marker genes, novel genes were also identified as potential markers.

### 4.7. Validation of scRNA-seq Results by FISH and Confocal Imaging

*w^1118^* flies were bred and T1, T2, and T3 L3 discs were dissected as described above. The discs were fixed in 3.7% paraformaldehyde on ice for 30 min. Then, probe hybridization was completed according to the protocol provided by Molecular Instruments. The discs were first permeabilized in a detergent solution containing sodium dodecyl sulfate and Tween-20, before custom-designed probes for *Dll*, *dac*, and *lncRNA:CR33938* were hybridized to the fixed and permeabilized discs for 20 h at 37 °C. After several washes with 5X SSC-Tween-20, hairpins with different fluorophores for each probe were added and incubated for 16 h in darkness at room temperature. The discs then underwent another several washes with 5X SSC-Tween-20 and were mounted onto a Menzel-Glaser Superforst Plus microscope slide (Thermo Scientific, Waltham, MA, USA, #J1800AMNZ) with a Hydromount mounting medium (National Diagnostics, Charlotte, NC, USA, #HS-106) and a 22 × 50 mm Deckglaser microscope coverglass (VWR, Radnor, PA, USA, #630-1461). The mounts were visualized on a Leica SP8 confocal microscope and each sample was imaged every 0.25 μm along the *z*-axis. The confocal images were z-stacked and processed with Leica Application Suite software.

### 4.8. Construction of lncRNA:CR33938 Expression Vector for Expression Studies

Total RNA was extracted from *D. melanogaster* L3 discs using NucleoZOL (Macherey-Nagel, UK, #740404.200) following the manufacturer’s protocol. Following RNase-free DNaseI (Thermo Scientific #EN0521) treatment and DNaseI inactivation by EDTA, the purified RNA was subjected to cDNA generation using PrimeScript II (Takara, Japan, #RR036A). The cDNA concentration was measured using the Qubit High Sensitivity double-stranded DNA assay. *lncRNA:CR33938* was amplified with PCR from the cDNA using the following primers with restriction site sequences inserted: forward primer 5′-TTTGGTACCTTGAGTCCGAGAGGTT-3′ and reverse primer 5′-CGCTCTAGACTCTTTTTTTGGTAGCCTATT-3′). The amplicon and the pAc5.1/V5-His B expression vector (Invitrogen, Waltham, MA, USA, #V411020) were digested with KpnI (New England Biolabs, USA, #R3142) and XbaI (New England Biolabs, Ipswich, MA, USA, #R0145) restriction enzymes and subsequently ligated using T4 DNA ligase (Invitrogen #15224017). The ligation mixture was transformed to chemically competent *Escherichia coli* (Invitrogen, #C404003) and selected using 100 mg/mL of ampicillin. The sequence of the *lncRNA:CR33938* construct cloned into the expression vector was verified by Sanger sequencing at the Beijing Genomics Institute. Transfection-ready plasmid DNA was extracted using a Plasmid Miniprep kit (Invitrogen, #K210011).

### 4.9. S2 Cell Culture and Transfection

*D. melanogaster* S2 cells were provided by Prof. Jerome Hui from the School of Life Sciences of The Chinese University of Hong Kong. S2 cells were cultured in Schneider’s Drosophila Medium (Gibco #21720) supplemented with 10% heat-inactivated fetal bovine serum (Gibco #10270) and 1% penicillin-streptomycin antibiotic mixtures (Gibco #15140122) in a 25 °C humidified incubator. The cloned pAc5.1-lncRNA:CR33938 construct was transfected into S2 cells using Effectene (Qiagen, Germantown, TN, USA, #301425) and the pAc5.1 backbone vector was used as a negative control. The cells were then incubated at 25 °C for 48 h prior to RNA extraction for qRT-PCR.

### 4.10. RNA Extraction and qRT-PCR of S2 Cells

RNA was extracted with NucleoZOL (Macherey-Nagel, Allentown, PA, USA, #740404.200). Following RNase-free DNaseI (Thermo Scientific #EN0521) treatment and DNaseI inactivation by EDTA, the purified RNA was subjected to cDNA generation using PrimeScript II (Takara, Tokyo, Japan, #RR036A). The cDNA concentration was measured with a Qubit High Sensitivity double-stranded DNA assay. For qRT-PCR, 1 ng of template cDNA and 1xTB Green II (Takara #RR820) were added to each well of a 96-well plate (Axygen, Union City, CA, USA, #PCR-96-FSC) and covered with an optical adhesive film (Applied Biosystems ABI, Waltham, MA, USA, #4311971) prior to execution on a BioRad CFX96 real-time PCR detection system. Primer sequences for each tested gene are listed in Table S2.

### 4.11. Nuclei Isolation for scATAC-seq

The same suspension of single cells used for scRNA-seq was used for nuclei isolation for scATAC-seq. Nuclei isolation was performed according to a 10× Genomics low input protocol for scATAC-seq with some optimizations. The cell suspension was pelleted and lysed on ice in a buffer containing the detergents Tween-20 and nonidet-P40 (NP40) for 30 s. The isolated nuclei were then washed twice and resuspended in chilled 10× diluted nuclei buffer (provided by 10× Genomics). Trypan blue stained nuclei were observed under the microscope to assess nuclei quality.

### 4.12. DNA Library Preparation and scATAC-seq

DNA library preparation was performed according to the scATAC-seq preparation protocol (v1.1) of 10× Genomics. First, the nuclei suspensions were incubated in a transposition mix that included a transposase that preferentially fragments the DNA in open regions of the chromatin. Simultaneously, adapter sequences were added to the ends of the DNA fragments. As in scRNA-seq, a microfluidics chip was used to produce GEMs (Gel Bead-in-Emulsions), but in this case, the droplets contained a single microbead with attached sequences consisting of a unique cell barcode, a single nucleus, and DNA amplification reagents. Once the DNA from each nucleus was barcoded, all nuclei were pooled for DNA library construction. Because only the histone unbound areas of the genome are cut by the transposase, the library consisted of DNA fragments that represented the open chromatin regions of the genome. Quality control and qualitative analysis of the final library were performed on an Agilent Bioanalyzer High Sensitivity DNA chip. The libraries were sequenced on the Illumina NovaSeq6000 platform by Novogene at PE50 with a sequencing depth of approximately 50,000 read pairs per cell.

### 4.13. scATAC-seq Data Analysis

The raw paired-end sequencing data were processed by Cell Ranger ATAC pipeline v2.0.0 with default settings, using a dm6 UCSC reference generated by the 10× Genomics mkref function. Data processing, filtering, dimensionality reduction, and clustering were performed with ArchR v1.0.1 [46]. UMAP visualizations of the scATAC-seq clusters were created before and after integration with scRNA-seq data. Determination of cell type identities were aided by manual annotation of cell type-specific marker genes based on gene scores estimated from the chromatin accessibility data. Peak calling with MACS2 v2.2.7.1 [47] was performed on each cell cluster. Identification of robust peak sets allowed the prediction of enriched transcription factor motifs for each cluster. Gene ontology analysis was performed using Cluster Profiler to determine the enriched distal process in T2 and T3 relative to T1. Genome browser plots depicting co-accessibility of distal genes with nearby genes were also generated using ArchR.

## Data Availability

The sequencing data presented in this study are openly available in NCBI SRA with BioProject ID PRJNA831899.

## Supplementary Materials

The following supporting information can be downloaded at: https://www.mdpi.com/article/10.3390/ijms23126796/s1.

## Appendix A

**Table A1:** Relative expression levels of marker genes. The average log2FC expression levels of the marker genes displayed in Figure 1 and Figure 2 are tabulated here.

Cell Subtype	Gene	Percent Expressed	Normalized UMI Count	Seurat Scaled Normalized UMI Count
Medial	*al*	14.47507953	0.446617753	−0.38657561
Medial	*C15*	1.033934252	0.020802751	−0.414645183
Medial	*Dll*	75.95440085	5.128735566	0.44025229
Medial	*dac*	68.31919406	3.578504256	1.883267974
Medial	*tsh*	8.324496288	0.18788577	−0.980689638
Medial	*hth*	39.10392365	4.268092807	−0.888707309
Medial	*vg*	1.802757158	0.078396772	−0.412093522
Medial	*salr*	1.272534464	0.028393623	−0.448546528
Medial	*salm*	1.405090138	0.032101424	−0.457590917
Medial	*hui*	10.49840933	0.275768473	−0.441380335
Medial	*betaTub56D*	99.73488865	67.01346734	−0.603531241
Medial	*alphaTub84B*	99.70837752	57.70906617	−0.54154286
Medial	*tsr*	99.60233298	52.63663428	−0.473061117
Medial	*Act5C*	99.76139979	61.24349032	−0.509223741
Proximal	*al*	17.52711497	0.862922147	0.067037547
Proximal	*C15*	0.954446855	0.015783215	−0.4175847
Proximal	*Dll*	28.63340564	1.420345973	−0.645129847
Proximal	*dac*	18.13449024	0.723348582	−0.290659318
Proximal	*tsh*	71.32321041	4.083784238	1.609816265
Proximal	*hth*	96.52928416	79.60652398	1.461906241
Proximal	*vg*	1.561822126	0.068269738	−0.417599705
Proximal	*salr*	2.212581345	0.12027794	−0.360530445
Proximal	*salm*	2.993492408	0.195738255	−0.347537362
Proximal	*hui*	8.937093275	0.343389783	−0.426442771
Proximal	*betaTub56D*	99.65292842	67.46030873	−0.576381945
Proximal	*alphaTub84B*	99.52277657	59.83865741	−0.467998663
Proximal	*tsr*	99.43600868	53.94964304	−0.392002795
Proximal	*Act5C*	99.69631236	64.22054168	−0.447496433
Fate_Undetermined	*al*	7.564469914	0.479912329	−0.350297209
Fate_Undetermined	*C15*	1.088825215	0.054030437	−0.395186544
Fate_Undetermined	*Dll*	37.13467049	2.905723476	−0.210385285
Fate_Undetermined	*dac*	24.41260745	1.538342317	0.329880269
Fate_Undetermined	*tsh*	13.29512894	0.933420238	−0.484960231
Fate_Undetermined	*hth*	51.57593123	10.93855676	−0.680584009
Fate_Undetermined	*vg*	1.891117479	0.261258161	−0.312669703
Fate_Undetermined	*salr*	1.604584527	0.097676107	−0.382180764
Fate_Undetermined	*salm*	2.063037249	0.167802628	−0.3663254
Fate_Undetermined	*hui*	6.074498567	0.405081245	−0.412815111
Fate_Undetermined	*betaTub56D*	99.94269341	87.40336921	0.635323338
Fate_Undetermined	*alphaTub84B*	99.88538682	77.19868569	0.131519833
Fate_Undetermined	*tsr*	99.82808023	67.62449968	0.452211633
Fate_Undetermined	*Act5C*	99.77077364	82.08451192	−0.07709812
Proximal-Distal-Axis	*al*	10.36789298	0.323764339	−0.520439017
Proximal-Distal-Axis	*C15*	2.173913043	0.053608202	−0.395433811
Proximal-Distal-Axis	*Dll*	34.44816054	1.98972318	−0.478482888
Proximal-Distal-Axis	*dac*	12.04013378	0.424840171	−0.517944852
Proximal-Distal-Axis	*tsh*	29.59866221	2.366915062	0.468215741
Proximal-Distal-Axis	*hth*	81.27090301	59.52749037	0.835425868
Proximal-Distal-Axis	*vg*	39.96655518	4.585898734	2.038686595
Proximal-Distal-Axis	*salr*	43.81270903	2.625894433	2.039602193
Proximal-Distal-Axis	*salm*	53.34448161	3.74465689	2.039279287
Proximal-Distal-Axis	*hui*	6.18729097	0.155657069	−0.467912975
Proximal-Distal-Axis	*betaTub56D*	99.49832776	71.17380529	−0.350756424
Proximal-Distal-Axis	*alphaTub84B*	98.82943144	59.83141261	−0.468248858
Proximal-Distal-Axis	*tsr*	98.66220736	44.76118553	−0.959250307
Proximal-Distal-Axis	*Act5C*	99.49832776	55.6216358	−0.625789399
Distal	*al*	60	2.603285376	1.963370297
Distal	*C15*	73.57142857	4.214292742	2.041126432
Distal	*Dll*	92.14285714	9.809431808	1.810211936
Distal	*dac*	9.642857143	0.250736492	−0.650508111
Distal	*tsh*	9.642857143	0.248253186	−0.940549437
Distal	*hth*	27.85714286	2.856922579	−0.932736841
Distal	*vg*	1.071428571	0.02411907	−0.441604926
Distal	*salr*	1.071428571	0.013810745	−0.462515482
Distal	*salm*	2.142857143	0.045931453	−0.448289565
Distal	*hui*	12.85714286	0.966716888	−0.288749653
Distal	*betaTub56D*	100	62.93114147	−0.851566182
Distal	*alphaTub84B*	100	55.43422202	−0.620103282
Distal	*tsr*	100	53.09832627	−0.444558658
Distal	*Act5C*	100	69.10840865	−0.346149551
Anterior-Posterior-Axis	*al*	4.519774011	0.091887873	−0.773096008
Anterior-Posterior-Axis	*C15*	1.129943503	0.014602416	−0.418276194
Anterior-Posterior-Axis	*Dll*	13.55932203	0.493279925	−0.916466206
Anterior-Posterior-Axis	*dac*	5.649717514	0.11476684	−0.754035963
Anterior-Posterior-Axis	*tsh*	56.49717514	2.156294232	0.3281673
Anterior-Posterior-Axis	*hth*	88.70056497	39.31225982	0.204696051
Anterior-Posterior-Axis	*vg*	0	0	−0.454718739
Anterior-Posterior-Axis	*salr*	1.694915254	0.093867563	−0.385828973
Anterior-Posterior-Axis	*salm*	3.389830508	0.088684597	−0.419536042
Anterior-Posterior-Axis	*hui*	79.0960452	11.49658469	2.037300845
Anterior-Posterior-Axis	*betaTub56D*	100	105.6986505	1.746912454
Anterior-Posterior-Axis	*alphaTub84B*	100	130.3298526	1.966373831
Anterior-Posterior-Axis	*tsr*	100	89.72629303	1.816661244
Anterior-Posterior-Axis	*Act5C*	100	182.5387119	2.005757244

**Table A2:** DEGs of fate undetermined cells. DEG list generated with cutoff value of log2FC equals 0.25 and minimum percentage of cells expressing the DEG of 0.25.

Gene	p_val	avg_log2FC	pct.1	pct.2
*eEF2*	2.63 × 10^−76^	0.541911833	0.949	0.968
*fabp*	1.12 × 10^−11^	0.468294279	0.655	0.722
*Uba1*	1.76 × 10^−5^	0.462953007	0.446	0.655
*CG14984*	0.000107	0.459747394	0.323	0.445
*Stip1*	2.15 × 10^−8^	0.417890064	0.375	0.582
*Karybeta3*	5.45 × 10^−13^	0.411510198	0.293	0.469
*CG3226*	0.024048	0.409442343	0.553	0.731
*SmD3*	5.37 × 10^−21^	0.403214155	0.733	0.868
*Ran*	4.25 × 10^−44^	0.394201883	0.888	0.942
*AsnRS*	1.47 × 10^−11^	0.392768286	0.36	0.569
*Nph*	1.83 × 10^−39^	0.388031758	0.86	0.928
*CG9922*	0.673966	0.381062842	0.531	0.76
*UQCR-Q*	1.16 × 10^−23^	0.377240451	0.782	0.901
*SmD2*	2.31 × 10^−16^	0.375790159	0.724	0.865
*eIF3b*	0.002117	0.374134239	0.619	0.813
*CCT2*	0.275826	0.370703182	0.539	0.763
*CG8149*	5.72 × 10^−7^	0.369303556	0.41	0.627
*COX5A*	2.80 × 10^−27^	0.36834579	0.789	0.9
*CG3760*	4.05 × 10^−6^	0.3672513	0.625	0.792
*san*	0.000989	0.366350781	0.443	0.663
*CCT7*	0.000414	0.36533304	0.601	0.799
*Tudor-SN*	0.001026	0.363468674	0.489	0.701
*CG11858*	0.594245	0.362893992	0.531	0.743
*CG11980*	8.49 × 10^−10^	0.362794172	0.382	0.598
*Fkbp39*	1.10 × 10^−34^	0.362430772	0.837	0.917
*CG17202*	0.063868	0.362292286	0.508	0.74
*Cyt-c-p*	0.739486	0.360850946	0.547	0.754
*Tim9a*	8.62 × 10^−5^	0.360541648	0.447	0.675
*UQCR-14*	5.71 × 10^−9^	0.35959829	0.693	0.862
*Rpb8*	0.014119	0.3594748	0.474	0.699
*CCT8*	3.72 × 10^−8^	0.355410141	0.642	0.81
*alphaTub84B*	9.72 × 10^−106^	0.351533668	0.999	0.996
*ATPsynbeta*	5.99E-54	0.351328277	0.926	0.955
*betaTub56D*	2.12 × 10^−111^	0.351110198	0.999	0.997
*eEF1delta*	3.35 × 10^−36^	0.348262336	0.861	0.934
*eIF6*	8.97 × 10^−5^	0.346187749	0.437	0.661
*CG11267*	4.58 × 10^−18^	0.345982895	0.788	0.902
*Aos1*	3.11 × 10^−5^	0.345697042	0.633	0.808
*NHP2*	1.59 × 10^−5^	0.34467569	0.66	0.835
*mod*	1.41 × 10^−12^	0.343973569	0.709	0.863
*CG2021*	4.20 × 10^−12^	0.343366272	0.345	0.547
*Nurf-38*	5.99 × 10^−18^	0.339601891	0.75	0.879
*ND-ACP*	9.76 × 10^−13^	0.338718622	0.711	0.872
*CG13994*	1.07 × 10^−15^	0.337794285	0.295	0.484
*tsr*	1.61 × 10^−102^	0.336728868	0.998	0.995
*ATPsynCF6*	1.79 × 10^−26^	0.335348459	0.818	0.923
*Nedd8*	1.67 × 10^−12^	0.334310017	0.715	0.876
*Nap1*	6.07 × 10^−12^	0.334107702	0.773	0.895
*hoip*	6.18 × 10^−6^	0.333796151	0.689	0.847
*SmB*	1.14 × 10^−20^	0.333628184	0.785	0.895
*Incenp*	2.62 × 10^−15^	0.332481608	0.315	0.513
*CG14210*	6.88 × 10^−10^	0.332309093	0.381	0.598
*alpha-Spec*	7.27 × 10^−22^	0.331563515	0.298	0.518
*Act5C*	6.76 × 10^−84^	0.330948608	0.998	0.997
*Pfdn5*	0.000266	0.328369305	0.65	0.83
*tsu*	0.026133	0.32834557	0.602	0.804
*RanGAP*	1.02 × 10^−11^	0.328311508	0.339	0.531
*CCT1*	0.001893	0.326762196	0.621	0.814
*CRIF*	8.74 × 10^−14^	0.326369065	0.313	0.499
*Ahcy*	0.140383	0.325984833	0.497	0.717
*UbcE2M*	2.61 × 10^−5^	0.324931312	0.452	0.684
*LysRS*	1.92 × 10^−20^	0.324481333	0.284	0.486
*Pfdn1*	0.015668	0.322437535	0.505	0.745
*janA*	0.027436	0.322236117	0.64	0.84
*Ote*	2.27 × 10^−11^	0.321707361	0.335	0.517
*Rpn9*	0.001611	0.321592929	0.626	0.817
*CG1542*	1.21 × 10^−12^	0.321248406	0.351	0.568
*COX5B*	1.43 × 10^−14^	0.320658424	0.768	0.899
*eIF3l*	3.71 × 10^−6^	0.320621493	0.434	0.656
*Pen*	3.70 × 10^−15^	0.320548585	0.764	0.892
*nop5*	3.23 × 10^−8^	0.31963021	0.432	0.655
*HP4*	0.192532	0.319387871	0.517	0.737
*NAT1*	1.18 × 10^−16^	0.318248392	0.38	0.613
*Rpn1*	1.24 × 10^−10^	0.318033663	0.422	0.661
*ND-51*	1.15 × 10^−9^	0.31799648	0.41	0.652
*Txl*	0.013031	0.317304773	0.484	0.704
*Prosbeta5*	8.73 × 10^−19^	0.316609822	0.779	0.894
*wac*	0.050386	0.316434419	0.484	0.703
*Hsc70-4*	5.19 × 10^−63^	0.316015021	0.982	0.986
*me31B*	0.419468	0.315758713	0.581	0.797
*GluProRS*	1.96 × 10^−9^	0.315644484	0.181	0.28
*Prp8*	1.00 × 10^−14^	0.3153532	0.221	0.363
*Set*	1.77 × 10^−13^	0.3136136	0.755	0.882
*Pfdn2*	7.64 × 10^−6^	0.313432961	0.669	0.849
*SelD*	0.008104	0.313112222	0.61	0.807
*HmgD*	3.22 × 10^−58^	0.31212012	0.995	0.994
*Jafrac1*	1.11 × 10^−22^	0.311403095	0.867	0.935
*Mcm6*	4.23 × 10^−10^	0.309433046	0.267	0.404
*lost*	0.001046	0.309298263	0.7	0.873
*Sem1*	0.001049	0.309268908	0.67	0.863
*smid*	2.02 × 10^−16^	0.308262181	0.306	0.505
*CG15019*	0.475046	0.308257341	0.587	0.808
*Echs1*	0.000121	0.308112475	0.662	0.836
*CG34132*	4.59 × 10^−11^	0.307967688	0.386	0.61
*Rpn10*	0.557285	0.30744727	0.577	0.784
*CCT5*	1.39 × 10^−12^	0.306858295	0.739	0.862
*baf*	1.48 × 10^−9^	0.30568901	0.746	0.88
*CG16985*	7.99 × 10^−12^	0.305096172	0.231	0.368
*ND-13B*	0.003083	0.304167028	0.516	0.768
*Cdk1*	0.182413	0.3034567	0.507	0.731
*Phb2*	0.056628	0.30342253	0.597	0.798
*La*	0.012425	0.30287118	0.513	0.741
*Prp19*	5.29E-15	0.301071904	0.342	0.558
*CG12321*	4.27E-21	0.300344146	0.292	0.501
*ox*	0.214652	0.299991914	0.533	0.769
*Prosalpha3*	5.63E-19	0.298876442	0.791	0.903
*SmE*	4.45E-06	0.298829465	0.694	0.867
*CG3594*	2.93E-15	0.298465681	0.286	0.471
*Pomp*	5.66E-19	0.298305603	0.813	0.907
*CG5903*	0.6842	0.297434191	0.571	0.795
*Acbp1*	0.059744	0.297149608	0.557	0.768
*CG6523*	0.022022	0.296185019	0.602	0.808
*aurB*	3.70E-05	0.295450632	0.4	0.586
*ND-SGDH*	0.175992	0.295209903	0.581	0.802
*mtSSB*	1.81E-10	0.294435499	0.409	0.639
*Rpb5*	1.51E-06	0.293998942	0.444	0.68
*CG2862*	8.93E-22	0.293161476	0.83	0.931
*Sgt*	0.056897	0.292139314	0.537	0.763
*pch2*	9.67E-12	0.292049575	0.399	0.631
*CG12848*	7.97E-15	0.292017665	0.334	0.547
*His2Av*	2.63E-27	0.291847689	0.915	0.954
*Prosalpha7*	0.000855	0.290548841	0.652	0.843
*ATPsynD*	1.62E-27	0.289427165	0.868	0.941
*CCT3*	4.81E-05	0.289356898	0.669	0.852
*tko*	0.060424	0.288967965	0.515	0.744
*ND-19*	6.71E-06	0.288617644	0.468	0.717
*CG9752*	1.15E-05	0.288354586	0.316	0.44
*mge*	2.15E-08	0.288185938	0.449	0.704
*Srp19*	8.31E-13	0.287931596	0.401	0.64
*Chc*	3.98E-16	0.287462202	0.377	0.612
*eIF3j*	6.29E-20	0.287256028	0.367	0.623
*CG14543*	1.82E-16	0.286760991	0.276	0.458
*CCT6*	0.088352	0.286307611	0.598	0.809
*Mdh2*	2.50E-12	0.285312875	0.39	0.639
*bonsai*	2.32E-18	0.284517765	0.311	0.53
*feo*	2.85E-14	0.28445883	0.342	0.551
*CG5355*	7.68E-19	0.284380697	0.371	0.611
*GlyRS*	6.75E-13	0.283564199	0.405	0.644
*CG9205*	2.12E-11	0.28348611	0.394	0.623
*Rrp40*	5.61E-18	0.282892518	0.274	0.461
*l(1)G0004*	1.37E-17	0.282407454	0.303	0.511
*Non2*	9.28E-23	0.282276666	0.856	0.941
*CG10576*	3.19E-07	0.281613969	0.695	0.85
*CG6617*	1.29E-11	0.281332974	0.408	0.653
*CCT4*	3.04E-07	0.280798696	0.7	0.856
*Tom7*	1.14E-06	0.280078089	0.706	0.878
*Pfdn6*	0.957133	0.279903383	0.564	0.792
*Uch*	1.44E-06	0.279613571	0.492	0.738
*CG4866*	1.67E-13	0.279468463	0.341	0.555
*roh*	9.19E-20	0.279380844	0.816	0.916
*Rpn13*	0.12711	0.279322366	0.543	0.772
*alphaTub84D*	3.38E-07	0.279114348	0.666	0.844
*Nlp*	3.51E-44	0.278341613	0.955	0.978
*fzy*	1.92E-16	0.277611464	0.293	0.481
*Nmt*	1.32E-15	0.277587163	0.364	0.6
*CG34200*	2.80E-19	0.277548087	0.342	0.573
*CG3420*	6.50E-15	0.2771143	0.362	0.589
*Cdc37*	9.32E-12	0.276940009	0.45	0.704
*CG10038*	6.04E-16	0.276910568	0.308	0.506
*CG10638*	8.48E-05	0.276901373	0.466	0.701
*Tim23*	0.098321	0.27545809	0.536	0.772
*Rpn11*	0.777644	0.274812562	0.548	0.771
*CG5515*	8.66E-09	0.274683335	0.434	0.676
*Pcd*	9.01E-12	0.274174524	0.433	0.671
*DENR*	7.60E-11	0.27411005	0.418	0.67
*CG8635*	4.18E-08	0.274072152	0.449	0.698
*Rpn12*	0.021678	0.273993176	0.513	0.745
*pont*	1.01E-08	0.273709261	0.417	0.654
*Grx1*	6.78E-20	0.273641436	0.344	0.58
*Gart*	6.00E-18	0.27314057	0.297	0.5
*Nacalpha*	6.76E-49	0.272817196	0.981	0.988
*GstO2*	4.65E-08	0.272726982	0.441	0.685
*CG14817*	6.98E-14	0.272320839	0.377	0.598
*cype*	7.38E-26	0.2714151	0.857	0.948
*COX7A*	2.08E-20	0.270870929	0.858	0.936
*Rpn8*	5.05E-07	0.270666209	0.468	0.725
*Prosalpha2*	1.35E-06	0.27064609	0.708	0.865
*Prosalpha5*	4.93E-09	0.270487893	0.705	0.86
*Rpn7*	0.494035	0.27046284	0.551	0.765
*Ski6*	1.25E-18	0.270029048	0.269	0.461
*Scsalpha1*	0.33089	0.269419769	0.568	0.784
*COX8*	2.60E-14	0.269389489	0.808	0.923
*CG7630*	1.15E-13	0.268873423	0.803	0.922
*Arp1*	2.34E-15	0.268500605	0.411	0.662
*Prosbeta7*	0.700295	0.268425349	0.597	0.804
*eIF3c*	6.27E-06	0.268191638	0.509	0.751
*CG6937*	1.25E-14	0.267816225	0.356	0.588
*Rae1*	8.51E-20	0.267383088	0.301	0.509
*Art1*	0.001476	0.266939135	0.488	0.72
*Roc1a*	6.33E-16	0.266631796	0.801	0.918
*CG17059*	2.17E-09	0.266613285	0.453	0.708
*Non3*	4.49E-17	0.266495993	0.339	0.564
*ncd*	4.47E-16	0.264327838	0.277	0.447
*CG4511*	1.92E-16	0.264239389	0.394	0.649
*Updo*	2.45E-20	0.264041525	0.345	0.591
*CG1598*	1.46E-17	0.263529104	0.362	0.604
*CG1789*	3.81E-22	0.263440367	0.319	0.548
*ATPsynE*	8.47E-21	0.263433084	0.848	0.943
*Nop56*	0.013911	0.262395693	0.655	0.841
*Prosbeta6*	0.000207	0.262237698	0.678	0.857
*COX4*	1.30E-19	0.262022839	0.844	0.936
*CG13364*	5.33E-09	0.261858812	0.768	0.914
*Rpt5*	1.59E-07	0.26115752	0.467	0.709
*CG9667*	8.35E-18	0.261015475	0.269	0.454
*ATPsynB*	1.97E-20	0.261004129	0.854	0.934
*ND-13A*	1.30E-15	0.260501665	0.389	0.632
*Ssb-c31a*	0.428003	0.260444449	0.609	0.813
*eEF1beta*	2.95E-47	0.260441246	0.986	0.989
*RnrL*	3.99E-14	0.25996256	0.231	0.365
*ND-B15*	0.00029	0.259827447	0.501	0.742
*p23*	3.03E-21	0.259045322	0.88	0.947
*RFeSP*	0.446974	0.258835685	0.601	0.82
*AIMP2*	1.69E-19	0.258671195	0.332	0.569
*CG17776*	1.54E-13	0.257691639	0.398	0.638
*Usp5*	4.82E-22	0.257589507	0.232	0.413
*Rpn3*	9.87E-05	0.257434516	0.487	0.726
*blw*	1.28E-26	0.257118864	0.883	0.946
*Rpn5*	0.209107	0.256465518	0.572	0.8
*pAbp*	3.06E-12	0.256184891	0.972	0.994
*ssx*	6.04E-19	0.256144854	0.193	0.338
*CHORD*	3.20E-17	0.255801888	0.191	0.33
*Cbs*	1.29E-15	0.255282574	0.308	0.498
*Vha14-1*	0.000131	0.255064003	0.497	0.74
*CG9643*	2.04E-22	0.254773967	0.286	0.495
*Fib*	2.20E-09	0.254765817	0.444	0.685
*endos*	0.001143	0.2546236	0.677	0.86
*msd5*	3.63E-13	0.254565067	0.268	0.423
*Prosbeta3*	8.21E-15	0.254528661	0.785	0.904
*cl*	0.010449	0.254513723	0.679	0.86
*CG11444*	0.171588	0.25436775	0.559	0.779
*CG8891*	8.66E-09	0.254175553	0.443	0.691
*icln*	0.000109	0.25393175	0.492	0.748
*mAcon1*	2.12E-21	0.253581166	0.295	0.51
*SmF*	0.001047	0.253472919	0.677	0.866
*porin*	1.68E-18	0.252948112	0.846	0.943
*Nup50*	1.92E-19	0.252683608	0.259	0.443
*dpa*	1.96E-08	0.252625619	0.22	0.322
*thoc6*	1.31E-18	0.252359096	0.296	0.498
*RpA-70*	4.90E-19	0.252331257	0.243	0.413
*CG9705*	0.019547	0.252183496	0.676	0.873
*Shmt*	1.73E-06	0.251894976	0.455	0.699
*alien*	3.33E-15	0.251507249	0.385	0.636
*Arl2*	1.16E-21	0.251458627	0.256	0.445
*SerRS*	0.076293	0.250933288	0.547	0.774
*CG1943*	2.38E-10	0.250821203	0.795	0.895
*ND-B14.5B*	5.78E-05	0.250452901	0.496	0.755

**Table A3:** Gene ontology analysis of the fate undetermined DEGs indicated enrichment in metabolic processes, DNA replication, RNA splicing and translation initiation suggestive of highly active, premature, growing cells.

Analysis Type:	PANTHER Overrepresentation Test (Released on 2 February 2022)						
Annotation Version and Release Date:	GO Ontology database DOI: 10.5281/zenodo.6399963 Released on 22 March 2022						
Analyzed List:	upload_1 (Drosophila melanogaster)						
Reference List:	Drosophila melanogaster (all genes in database)						
Test Type:	FISHER						
Correction:	FDR						
GO biological process complete	Drosophila melanogaster—REFLIST (13821)	upload_1 (205)	upload_1 (expected)	upload_1 (over/under)	upload_1 (fold Enrichment)	upload_1 (raw P-value)	upload_1 (FDR)
mitochondrial electron transport, ubiquinol to cytochrome c (GO:0006122)	13	5	0.19	+	25.93	4.66E-06	4.54E-04
DNA unwinding involved in DNA replication (GO:0006268)	11	4	0.16	+	24.52	5.33E-05	4.03E-03
spliceosomal snRNP assembly (GO:0000387)	18	6	0.27	+	22.47	9.79E-07	1.12E-04
proton motive force-driven ATP synthesis (GO:0015986)	21	6	0.31	+	19.26	2.08E-06	2.25E-04
mitochondrial electron transport, cytochrome c to oxygen (GO:0006123)	15	4	0.22	+	17.98	1.45E-04	9.39E-03
ATP biosynthetic process (GO:0006754)	23	6	0.34	+	17.59	3.25E-06	3.38E-04
purine ribonucleoside triphosphate biosynthetic process (GO:0009206)	27	6	0.4	+	14.98	7.22E-06	6.54E-04
purine ribonucleoside triphosphate metabolic process (GO:0009205)	27	6	0.4	+	14.98	7.22E-06	6.47E-04
purine nucleoside triphosphate biosynthetic process (GO:0009145)	27	6	0.4	+	14.98	7.22E-06	6.39E-04
aerobic electron transport chain (GO:0019646)	54	12	0.8	+	14.98	1.70E-10	4.56E-08
purine nucleoside triphosphate metabolic process (GO:0009144)	28	6	0.42	+	14.45	8.66E-06	7.42E-04
mitochondrial electron transport, NADH to ubiquinone (GO:0006120)	19	4	0.28	+	14.19	3.16E-04	1.89E-02
mitochondrial ATP synthesis coupled electron transport (GO:0042775)	58	12	0.86	+	13.95	3.48E-10	7.34E-08
ribonucleoside triphosphate biosynthetic process (GO:0009201)	30	6	0.44	+	13.48	1.22E-05	1.03E-03
ribonucleoside triphosphate metabolic process (GO:0009199)	30	6	0.44	+	13.48	1.22E-05	1.01E-03
nucleoside triphosphate metabolic process (GO:0009141)	36	7	0.53	+	13.11	2.65E-06	2.83E-04
ATP synthesis coupled electron transport (GO:0042773)	62	12	0.92	+	13.05	6.83E-10	1.40E-07
respiratory electron transport chain (GO:0022904)	69	13	1.02	+	12.7	1.75E-10	4.54E-08
oxidative phosphorylation (GO:0006119)	69	13	1.02	+	12.7	1.75E-10	4.39E-08
electron transport chain (GO:0022900)	76	14	1.13	+	12.42	4.43E-11	1.38E-08
nucleoside triphosphate biosynthetic process (GO:0009142)	33	6	0.49	+	12.26	1.98E-05	1.60E-03
germarium-derived female germ-line cyst formation (GO:0030727)	23	4	0.34	+	11.73	5.97E-04	3.35E-02
female germ-line cyst formation (GO:0048135)	24	4	0.36	+	11.24	6.89E-04	3.75E-02
DNA duplex unwinding (GO:0032508)	25	4	0.37	+	10.79	7.90E-04	4.16E-02
aerobic respiration (GO:0009060)	101	16	1.5	+	10.68	1.33E-11	4.70E-09
cellular respiration (GO:0045333)	111	17	1.65	+	10.33	4.82E-12	1.88E-09
ATP metabolic process (GO:0046034)	118	18	1.75	+	10.28	1.18E-12	5.73E-10
mitochondrial respiratory chain complex I assembly (GO:0032981)	36	5	0.53	+	9.36	3.10E-04	1.90E-02
NADH dehydrogenase complex assembly (GO:0010257)	36	5	0.53	+	9.36	3.10E-04	1.89E-02
translational initiation (GO:0006413)	53	7	0.79	+	8.9	2.58E-05	2.05E-03
tRNA aminoacylation for protein translation (GO:0006418)	38	5	0.56	+	8.87	3.89E-04	2.30E-02
energy derivation by oxidation of organic compounds (GO:0015980)	135	17	2	+	8.49	7.90E-11	2.28E-08
tRNA aminoacylation (GO:0043039)	41	5	0.61	+	8.22	5.35E-04	3.04E-02
amino acid activation (GO:0043038)	43	5	0.64	+	7.84	6.53E-04	3.61E-02
protein folding (GO:0006457)	132	15	1.96	+	7.66	3.85E-09	6.82E-07
proteasome-mediated ubiquitin-dependent protein catabolic process (GO:0043161)	217	24	3.22	+	7.46	1.08E-13	1.20E-10
proteasomal protein catabolic process (GO:0010498)	229	24	3.4	+	7.07	3.16E-13	2.74E-10
generation of precursor metabolites and energy (GO:0006091)	183	18	2.71	+	6.63	8.59E-10	1.67E-07
centrosome cycle (GO:0007098)	72	7	1.07	+	6.55	1.53E-04	9.85E-03
ribonucleoprotein complex assembly (GO:0022618)	117	11	1.74	+	6.34	2.72E-06	2.86E-04
modification-dependent macromolecule catabolic process (GO:0043632)	318	29	4.72	+	6.15	2.43E-14	4.74E-11
microtubule organizing center organization (GO:0031023)	77	7	1.14	+	6.13	2.25E-04	1.41E-02
ribonucleoprotein complex subunit organization (GO:0071826)	122	11	1.81	+	6.08	3.96E-06	3.90E-04
modification-dependent protein catabolic process (GO:0019941)	311	28	4.61	+	6.07	9.51E-14	1.23E-10
ubiquitin-dependent protein catabolic process (GO:0006511)	307	27	4.55	+	5.93	4.57E-13	2.97E-10
proteolysis involved in cellular protein catabolic process (GO:0051603)	332	28	4.92	+	5.69	4.22E-13	2.99E-10
cellular protein catabolic process (GO:0044257)	334	28	4.95	+	5.65	4.83E-13	2.90E-10
microtubule cytoskeleton organization involved in mitosis (GO:1902850)	85	7	1.26	+	5.55	3.94E-04	2.31E-02
protein catabolic process (GO:0030163)	343	28	5.09	+	5.5	8.82E-13	4.58E-10
nuclear transport (GO:0051169)	118	9	1.75	+	5.14	1.02E-04	6.99E-03
nucleocytoplasmic transport (GO:0006913)	118	9	1.75	+	5.14	1.02E-04	6.93E-03
establishment of protein localization to membrane (GO:0090150)	95	7	1.41	+	4.97	7.35E-04	3.95E-02
purine ribonucleotide biosynthetic process (GO:0009152)	96	7	1.42	+	4.92	7.79E-04	4.13E-02
ribonucleoprotein complex biogenesis (GO:0022613)	288	21	4.27	+	4.92	5.00E-09	8.65E-07
cellular macromolecule catabolic process (GO:0044265)	447	32	6.63	+	4.83	5.01E-13	2.79E-10
purine-containing compound biosynthetic process (GO:0072522)	112	8	1.66	+	4.82	3.77E-04	2.24E-02
cell population proliferation (GO:0008283)	122	8	1.81	+	4.42	6.43E-04	3.58E-02
rRNA metabolic process (GO:0016072)	168	11	2.49	+	4.41	6.48E-05	4.80E-03
mRNA splicing, via spliceosome (GO:0000398)	214	14	3.17	+	4.41	6.56E-06	6.08E-04
RNA splicing, via transesterification reactions with bulged adenosine as nucleophile (GO:0000377)	214	14	3.17	+	4.41	6.56E-06	6.01E-04
rRNA processing (GO:0006364)	153	10	2.27	+	4.41	1.41E-04	9.25E-03
nucleotide biosynthetic process (GO:0009165)	123	8	1.82	+	4.39	6.77E-04	3.71E-02
RNA splicing, via transesterification reactions (GO:0000375)	216	14	3.2	+	4.37	7.25E-06	6.35E-04
macromolecule catabolic process (GO:0009057)	494	32	7.33	+	4.37	6.16E-12	2.29E-09
nucleoside phosphate biosynthetic process (GO:1901293)	124	8	1.84	+	4.35	7.12E-04	3.85E-02
spindle organization (GO:0007051)	125	8	1.85	+	4.31	7.48E-04	3.99E-02
translation (GO:0006412)	304	19	4.51	+	4.21	2.72E-07	3.47E-05
protein-containing complex assembly (GO:0065003)	433	27	6.42	+	4.2	7.19E-10	1.44E-07
establishment of protein localization to organelle (GO:0072594)	179	11	2.66	+	4.14	1.11E-04	7.43E-03
RNA splicing (GO:0008380)	228	14	3.38	+	4.14	1.29E-05	1.06E-03
peptide biosynthetic process (GO:0043043)	310	19	4.6	+	4.13	3.61E-07	4.39E-05
meiotic cell cycle (GO:0051321)	215	13	3.19	+	4.08	3.09E-05	2.40E-03
mitotic cell cycle (GO:0000278)	399	24	5.92	+	4.06	1.33E-08	2.11E-06
meiotic cell cycle process (GO:1903046)	204	12	3.03	+	3.97	8.02E-05	5.63E-03
organonitrogen compound catabolic process (GO:1901565)	514	30	7.62	+	3.93	3.34E-10	7.64E-08
microtubule cytoskeleton organization (GO:0000226)	344	20	5.1	+	3.92	3.88E-07	4.65E-05
nuclear division (GO:0000280)	246	14	3.65	+	3.84	2.88E-05	2.27E-03
cell cycle (GO:0007049)	608	34	9.02	+	3.77	5.64E-11	1.69E-08
ribosome biogenesis (GO:0042254)	215	12	3.19	+	3.76	1.29E-04	8.49E-03
mitochondrion organization (GO:0007005)	235	13	3.49	+	3.73	7.34E-05	5.20E-03
amide biosynthetic process (GO:0043604)	350	19	5.19	+	3.66	2.03E-06	2.22E-04
organelle fission (GO:0048285)	259	14	3.84	+	3.64	4.92E-05	3.75E-03
protein-containing complex organization (GO:0043933)	527	28	7.82	+	3.58	9.82E-09	1.59E-06
mRNA metabolic process (GO:0016071)	344	18	5.1	+	3.53	6.19E-06	5.81E-04
mRNA processing (GO:0006397)	268	14	3.98	+	3.52	6.98E-05	4.99E-03
cell cycle process (GO:0022402)	513	26	7.61	+	3.42	8.53E-08	1.17E-05
mitotic cell cycle process (GO:1903047)	277	14	4.11	+	3.41	9.75E-05	6.72E-03
chromosome organization (GO:0051276)	476	24	7.06	+	3.4	3.06E-07	3.84E-05
spermatogenesis (GO:0007283)	264	13	3.92	+	3.32	2.21E-04	1.40E-02
peptide metabolic process (GO:0006518)	409	20	6.07	+	3.3	4.90E-06	4.71E-04
protein localization to organelle (GO:0033365)	249	12	3.69	+	3.25	4.63E-04	2.67E-02
cellular catabolic process (GO:0044248)	801	38	11.88	+	3.2	3.45E-10	7.48E-08
microtubule-based process (GO:0007017)	464	21	6.88	+	3.05	8.79E-06	7.44E-04
male gamete generation (GO:0048232)	310	14	4.6	+	3.04	2.97E-04	1.84E-02
ncRNA metabolic process (GO:0034660)	377	17	5.59	+	3.04	6.83E-05	4.97E-03
cellular nitrogen compound biosynthetic process (GO:0044271)	711	32	10.55	+	3.03	3.45E-08	5.07E-06
regulation of catabolic process (GO:0009894)	291	13	4.32	+	3.01	5.39E-04	3.04E-02
RNA processing (GO:0006396)	560	25	8.31	+	3.01	1.46E-06	1.63E-04
organic substance catabolic process (GO:1901575)	823	36	12.21	+	2.95	8.56E-09	1.42E-06
intracellular protein transport (GO:0006886)	328	14	4.87	+	2.88	5.12E-04	2.93E-02
cellular amide metabolic process (GO:0043603)	493	21	7.31	+	2.87	2.10E-05	1.69E-03
female gamete generation (GO:0007292)	628	26	9.31	+	2.79	3.36E-06	3.45E-04
gene expression (GO:0010467)	1120	46	16.61	+	2.77	3.36E-10	7.48E-08
catabolic process (GO:0009056)	932	38	13.82	+	2.75	1.91E-08	2.92E-06
cellular macromolecule biosynthetic process (GO:0034645)	496	20	7.36	+	2.72	6.96E-05	5.02E-03
sexual reproduction (GO:0019953)	931	37	13.81	+	2.68	5.75E-08	8.30E-06
germ cell development (GO:0007281)	713	28	10.58	+	2.65	3.63E-06	3.63E-04
cellular component biogenesis (GO:0044085)	1285	50	19.06	+	2.62	2.90E-10	6.85E-08
organonitrogen compound biosynthetic process (GO:1901566)	800	31	11.87	+	2.61	1.34E-06	1.52E-04
cellular process involved in reproduction in multicellular organism (GO:0022412)	852	33	12.64	+	2.61	5.89E-07	6.95E-05
RNA metabolic process (GO:0016070)	828	32	12.28	+	2.61	9.37E-07	1.09E-04
gamete generation (GO:0007276)	915	35	13.57	+	2.58	3.38E-07	4.18E-05
oogenesis (GO:0048477)	576	22	8.54	+	2.58	6.48E-05	4.76E-03
cellular protein metabolic process (GO:0044267)	1683	63	24.96	+	2.52	4.07E-12	1.67E-09
cellular component assembly (GO:0022607)	1109	41	16.45	+	2.49	8.82E-08	1.18E-05
nucleobase-containing compound metabolic process (GO:0006139)	1369	50	20.31	+	2.46	2.65E-09	4.79E-07
cytoskeleton organization (GO:0007010)	580	21	8.6	+	2.44	3.13E-04	1.89E-02
nucleic acid metabolic process (GO:0090304)	1105	40	16.39	+	2.44	1.96E-07	2.54E-05
reproductive process (GO:0022414)	1190	43	17.65	+	2.44	6.16E-08	8.57E-06
developmental process involved in reproduction (GO:0003006)	892	32	13.23	+	2.42	5.10E-06	4.84E-04
cellular nitrogen compound metabolic process (GO:0034641)	1801	64	26.71	+	2.4	2.20E-11	7.45E-09
heterocycle metabolic process (GO:0046483)	1451	51	21.52	+	2.37	7.34E-09	1.24E-06
cellular macromolecule metabolic process (GO:0044260)	2106	74	31.24	+	2.37	3.46E-13	2.70E-10
organelle organization (GO:0006996)	1721	60	25.53	+	2.35	2.20E-10	5.35E-08
multicellular organismal reproductive process (GO:0048609)	1041	36	15.44	+	2.33	3.50E-06	3.54E-04
macromolecule biosynthetic process (GO:0009059)	696	24	10.32	+	2.32	1.57E-04	1.00E-02
cellular aromatic compound metabolic process (GO:0006725)	1495	51	22.17	+	2.3	1.55E-08	2.42E-06
proteolysis (GO:0006508)	861	29	12.77	+	2.27	5.96E-05	4.46E-03
organic cyclic compound metabolic process (GO:1901360)	1558	51	23.11	+	2.21	5.89E-08	8.35E-06
cellular metabolic process (GO:0044237)	3877	126	57.51	+	2.19	7.75E-23	3.02E-19
cellular component organization or biogenesis (GO:0071840)	2778	88	41.2	+	2.14	2.76E-13	2.69E-10
macromolecule localization (GO:0033036)	874	27	12.96	+	2.08	4.25E-04	2.47E-02
cellular biosynthetic process (GO:0044249)	1178	36	17.47	+	2.06	4.31E-05	3.32E-03
reproduction (GO:0000003)	1421	43	21.08	+	2.04	7.99E-06	6.92E-04
cellular component organization (GO:0016043)	2616	79	38.8	+	2.04	8.68E-11	2.41E-08
organic substance biosynthetic process (GO:1901576)	1212	36	17.98	+	2	9.41E-05	6.55E-03
macromolecule metabolic process (GO:0043170)	3193	94	47.36	+	1.98	1.44E-12	6.23E-10
biosynthetic process (GO:0009058)	1239	36	18.38	+	1.96	1.15E-04	7.63E-03
nitrogen compound metabolic process (GO:0006807)	3671	106	54.45	+	1.95	3.62E-14	5.64E-11
protein metabolic process (GO:0019538)	2183	63	32.38	+	1.95	1.26E-07	1.67E-05
multicellular organism reproduction (GO:0032504)	1275	36	18.91	+	1.9	2.28E-04	1.42E-02
metabolic process (GO:0008152)	4548	128	67.46	+	1.9	1.15E-17	3.00E-14
organonitrogen compound metabolic process (GO:1901564)	2725	75	40.42	+	1.86	2.76E-08	4.14E-06
primary metabolic process (GO:0044238)	4039	109	59.91	+	1.82	1.30E-12	5.96E-10
cellular process (GO:0009987)	7306	188	108.37	+	1.73	2.61E-33	2.03E-29
organic substance metabolic process (GO:0071704)	4283	110	63.53	+	1.73	2.91E-11	9.45E-09
biological_process (GO:0008150)	11314	197	167.81	+	1.17	1.87E-09	3.47E-07
Unclassified (UNCLASSIFIED)	2507	8	37.19	−	0.22	1.87E-09	3.55E-07
