# Supplementary material for: Single-Cell Atlas of the Drosophila Leg Disc Identifies a Long Non-Coding RNA in Late Development
Source: Int J Mol Sci. 2022 Jun 18;23(12):6796. doi: 10.3390/ijms23126796 (PMC9224501; doi:10.3390/ijms23126796)
Supplement: Supplementary file 1 [file ijms-23-06796-s001.zip › Supplementary_legends_Drosophila_singlecell_w1118.pdf]

Supplementary Figures / Tables Legends

Table S1. Sequencing statistics among the T1, T2 and T3 samples. Sequencing statistics showed similar data quality amongst the three samples, including percentage mapped reads, percentage of mapped reads aligned to genes, number of cells and mean reads per cell.

Table S2. List of qRT-PCR primer sequences of *Drosophila* leg development genes.

Figure S1. Expression of muscle markers across T1, T2 and T3. Early muscle cells increased expression of late muscle cell marker *Fas2* overtime, in terms of expression level and the number of cells that expressed the gene. Late muscle cell marker *Mef2*, a skeletal muscle differentiation transcription factor, increased expression in the late muscle cell subcluster overtime, also in terms of expression level and the number of cells that expressed the gene. Color scale represents expression level.

Figure S2. Heatmap of top upregulated genes in the early and late muscle cells. A distinction in upregulated genes were observed in the early versus late muscle cells. Color scale represents expression level.

Figure S3. Heatmap of top upregulated genes in the early and late neuronal cells. A distinction in upregulated genes were observed in the early versus late neuronal cells. Color scale represents expression level.

Figure S4. Heatmap of top upregulated genes in the various immune cells. A distinction in upregulated genes were observed in the hemocytes and plasmatocytes versus glial cells. Color scale represents expression level.
