# Supplementary material for: Single-Cell Atlas of the Drosophila Leg Disc Identifies a Long Non-Coding RNA in Late Development
Source: Int J Mol Sci. 2022 Jun 18;23(12):6796. doi: 10.3390/ijms23126796 (PMC9224501; doi:10.3390/ijms23126796)
Supplement: Supplementary file 1 [file ijms-23-06796-s001.zip › Table S1.pdf]

	T1	T2	T3
Total number of reads	136,708,491	105,793,081	117,487,726
Number of mapped reads (%)	131,732,137 (96.4%)	102,923,413 (97.3%)	113,716,848 (96.8%)
Number of unmapped reads (%)	4,976,354 (3.6%)	2,869,668 (2.7%)	3,770,878 (3.2%)
Number of reads aligned to genes	120,438,184	92,190,389	102,157,323
Percentage of mapped reads aligned to genes	91.4%	89.6%	89.8%
Number of genes with >= 20 total UMI counts across cells	8,223	7,905	7,651
Number of cells	3,623	3,365	3,704
Mean reads per cell	37,734	26,921	31,719
