# Supplementary material for: Single-Cell Atlas of the Drosophila Leg Disc Identifies a Long Non-Coding RNA in Late Development
Source: Int J Mol Sci. 2022 Jun 18;23(12):6796. doi: 10.3390/ijms23126796 (PMC9224501; doi:10.3390/ijms23126796)
Supplement: Supplementary file 1 [file ijms-23-06796-s001.zip › Table S2.pdf]

**Primer Name**	**Primer sequence(5'to3')**
bnl-For	TACGGCTCGAAAGACTTCACC
bnl-Rev	CCATTCAGGGCCAGGTAGAAGA
boi-For	TCCGATAAGAACAACTCTGGACTG
boi-Rev	CTGCTGGTGGTAGTCCAGTC
CG30371-For	CGTAGGAACGAGCACACCATTC
CG30371-Rev	GTCCTGGTTGGTGACCACATT
CG32333-For	CCATCTGCTAGTTGACAGTCGTC
CG32333-Rev	CATTGCCGTTCAGGCTGCT
CG43444-Tet-For	TGGACAAATTTCCCTGCGAGGTA
CG43444-Tet-Rev	GCTGCTCTGGCTATTGGCATC
CG6841-For	ATTGATGATGCTGAATTCTGCGCAA
CG6841-Rev	GGTTCTTTTCAAAGTAGGCGGCT
CG9129-For	AGAACGGAAGCTGTGTCAACC
CG9129-Rev	CTCTCCGTGCGCTTCTTGTAAA
chinmo-For	ACAGTCCAGTCGCTACGAGAAC
chinmo-Rev	CGGTCGTCGTCATTAGCCTCA
Cka-For	ATTGCAGGCTCGCATTGCTA
Cka-Rev	GGATCGGTGCCGTATTTTAAACG
CSN1b-For	CACCACTCATGCAGAACGC
CSN1b-Rev	CGATCCCGGCATATTGGTTG
disco-r-For	GATACTGGAGGTTGGCATCCACA
disco-r-Rev	GTCCAGCGGCATGCTATCAAT
eIF6-For	CAACACCTGCGTAACAGCCT
eIF6-Rev	ATCTCCTCGGTCTCCTTGTCCA
mys-For	TATCGCAAAATCTCCTCTTCGGT
mys-Rev	TCAGATTGTCGCACTTAGAGGTCT
PI31-For	CGTGGCGGATCCTTCATTCC
PI31-Rev	CCATATTTGGGCGTGATGGAAAG
Piezo-For	TGTTCTGACAACCGGACTGC
Piezo-Rev	CATGTAGATGATGTAGGTGCGCAA
sick-For	CAATGTTGACCACAAGTCCTCGA
sick-Rev	CGTGGTGCAGGTTGTCCAAA
Dll-For	TCCACCGAAGGATGACTTCTCC
Dll-Rev	CAGTTGCAGCGAGCTGTAGA
dac-For	GATGACGAAGATTTGAGTGACACG
dac-Rev	ACACCCAGATGGGACAGACC
hth-For	AAGGATGCGATTTATGAACATCCG
hth-Rev	CGATTCCGACGAACAGACATC
Hh-For	GCTCCGTCAAGTCAGATTCGTC
Hh-Rev	GTCATGCTCAAAACACGATCTCC
wg-For	GTCAGGGACGCAAGCATAATAGATA
wg-Rev	CTCGGCGAAGGCTCCAGATA
dpp-For	CACACAAAGATAGTAAAATCGACGA
dpp-Rev	ACCTGTTGACTGAGTGCGT
