# Supplementary figures and images for: Single-Cell Atlas of the Drosophila Leg Disc Identifies a Long Non-Coding RNA in Late Development
Source: Int J Mol Sci. 2022 Jun 18;23(12):6796. doi: 10.3390/ijms23126796 (PMC9224501; doi:10.3390/ijms23126796)

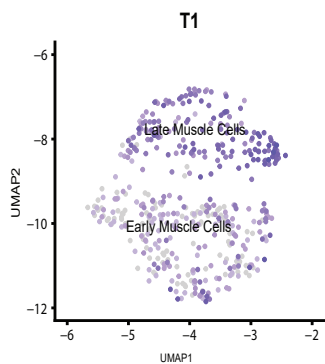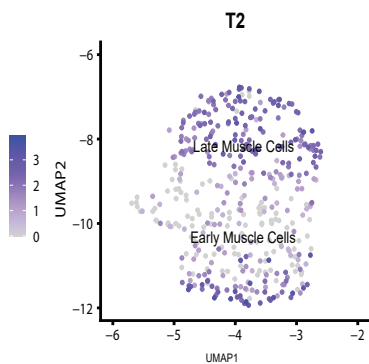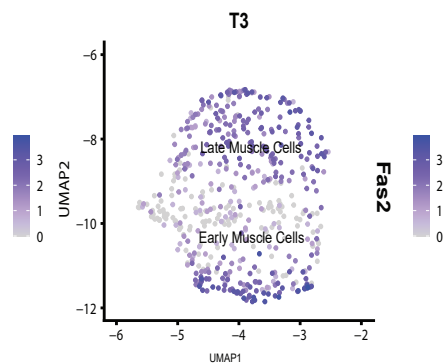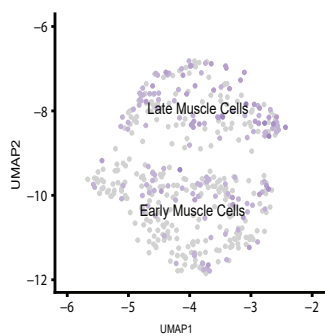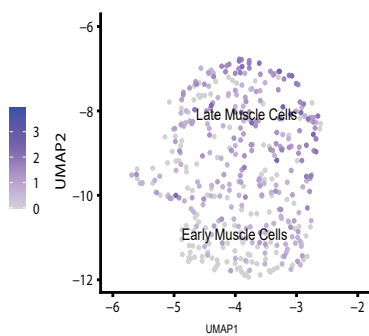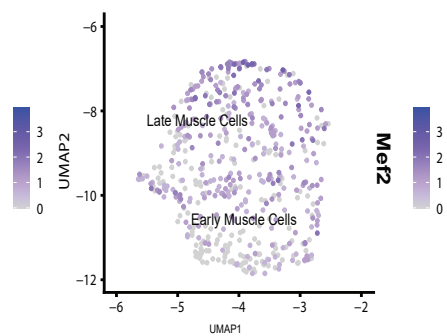

Supplement: Supplementary file 1 [file ijms-23-06796-s001.zip › Figure S1.pdf]

Early Muscle Cells

Late Muscle Cells

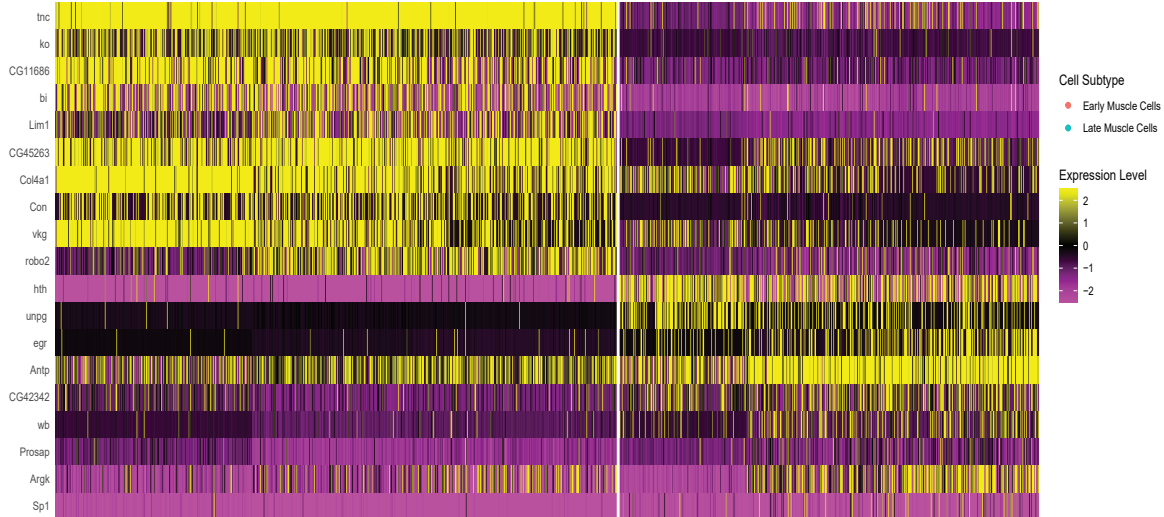

Supplement: Supplementary file 1 [file ijms-23-06796-s001.zip › Figure S2.pdf]

Early Neuronal Cells

Late Neuronal Cells

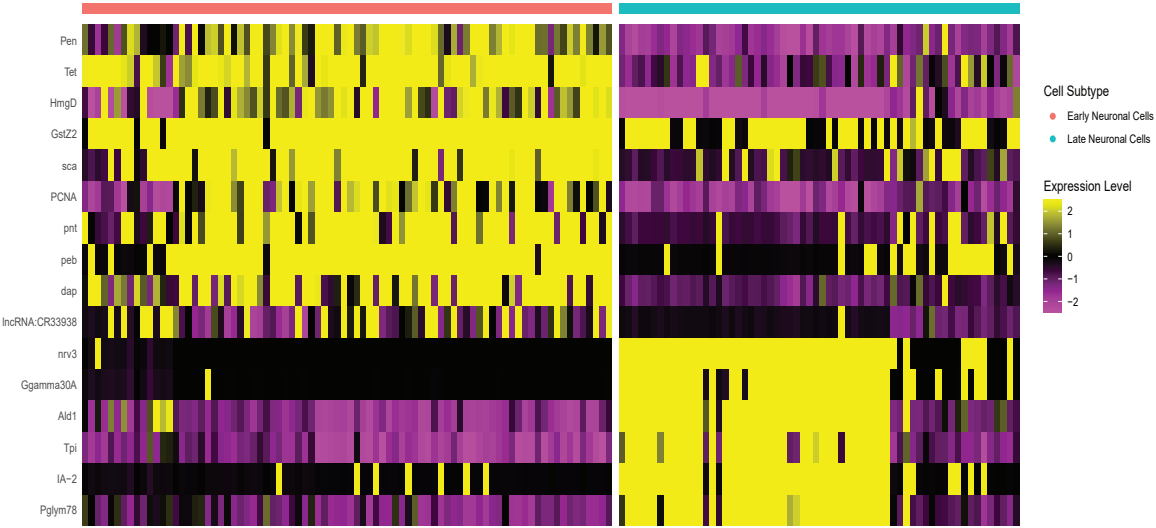

Supplement: Supplementary file 1 [file ijms-23-06796-s001.zip › Figure S3.pdf]

Hemocytes + Plasmatocytes

Glia

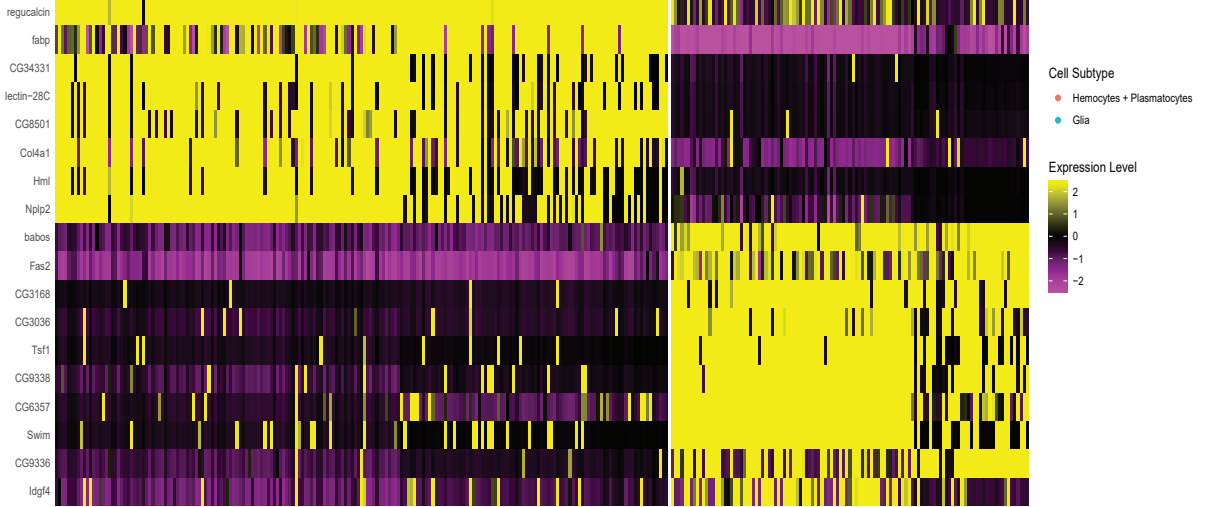

Supplement: Supplementary file 1 [file ijms-23-06796-s001.zip › Figure S4.pdf]
